# Fruit-stem structural visual perception for picking point localization in strawberry harvesting robots

**DOI:** 10.3389/fpls.2026.1894265

**Published:** 2026-07-29

**Authors:** Jiaxing Qing, Zhikang Zeng, Jinhong Chen, Xiaohe Liang, Bin Mo, Yawen Liu, Ailian Zhou, Saisai Wu

**Affiliations:** 1 College of Engineering, South China Agricultural University, Guangzhou, China; 2 Guangxi Academy of Agricultural Sciences, Nanning, China; 3 Agricultural Information Institute, Chinese Academy of Agricultural Sciences, Beijing, China; 4 Key Laboratory of Agricultural Blockchain Application, Ministry of Agriculture and Rural Affairs, Agricultural Information Institute, Chinese Academy of Agricultural Sciences, Beijing, China

**Keywords:** fruit-stem detection, oriented bounding box detection, picking point localization, strawberry harvesting robot, YOLOv11n-OBB

## Abstract

In ridge-cultivated strawberry environments, significant scale variation between fruits and stems, severe occlusion, and high missed-detection rates for small or slender targets pose major challenges to accurate picking point localization. This study proposes a fruit-stem oriented detection and picking point localization method based on an improved YOLOv11n-OBB model. Specifically, an enhanced model termed FSGE-OBB is developed. A Feature Fusion Lite Module (FFLM) is introduced to strengthen cross-layer interaction between shallow spatial details and deep semantic features, thereby improving multi-scale feature representation. A Stem Direction Enhancement Module (SDEM) is designed to enhance the directional perception of slender stems. In addition, Grouped Spatial Excitation Convolution (GSEConv) is adopted to reduce model complexity while maintaining feature representation capability, and an Efficient Upsampling Convolution Block (EUCB) is incorporated into high-resolution feature maps to improve detail recovery. Based on the oriented detection results, a geometry-constrained fruit-stem association method is established by modeling the spatial relationship between fruit and stem oriented bounding boxes (OBBs), and the corresponding picking points are localized accordingly. Experimental results show that the FSGE-OBB model achieves mAP@0.5 and mAP@0.5:0.95 of 78.85% and 68.27%, respectively, outperforming the YOLOv11n-OBB baseline by 2.59 and 1.90 percentage points. The proposed fruit-stem association method achieves a success rate of 97.99%. In preliminary three dimensional localization experiments, the mean absolute errors of picking points are 2.2 mm, 2.6 mm, and 2.3 mm in the *x-*, *y-*, and *z-*directions, respectively. Furthermore, simulated harvesting experiments achieve a success rate of 93.3%, with an average harvesting time of 9.2 s per fruit, validating the feasibility and effectiveness of the proposed method in practical harvesting tasks. These results demonstrate that the proposed FSGE-OBB-based framework enables unified modeling of fruit-stem detection, association, and picking point localization, providing a feasible visual perception solution for strawberry harvesting robots.

## 1 Introduction

Due to their high market value, strawberries are widely cultivated and in strong demand worldwide (Bitakou et al., 2026). China is one of the world’s major strawberry-producing countries (Li et al., 2026). At present, ridge cultivation remains one of the main planting methods for strawberries in China (Li et al., 2023). In ridge-cultivated environments, strawberry plants are relatively short, harvesting spaces are narrow, and fruits and stems are often occluded by leaves, which increases labor demand and reduces the efficiency of manual harvesting. Meanwhile, harvesting costs account for a considerable proportion of total production costs. Therefore, developing intelligent strawberry harvesting robots for ridge-cultivated scenarios can greatly enhance harvesting efficiency and advance the automation of the strawberry sector (Zhao et al., 2023).

Because the skin of ripe strawberries is soft and easily damaged during harvesting, strawberry harvesting robots usually adopt a stem-cutting strategy (Liu et al., 2026). In this study, the picking point refers to the stem-cutting position used by the harvesting end-effector. This harvesting strategy imposes higher requirements on the vision system, which must accurately detect ripe fruits, identify stems, and precisely localize picking points (Tituaña et al., 2024; Wang L, et al., 2026). Existing vision-based methods for strawberry fruit detection and picking point localization can generally be divided into keypoint detection-based methods, horizontal bounding box (HBB)-based methods, segmentation-based methods, and fruit oriented bounding box (OBB)-based methods. Keypoint detection-based methods can directly predict picking points on fruits or stems and usually achieve high localization accuracy when the fruit-stem region is clearly visible. However, their performance tends to degrade under severe occlusion or incomplete stem visibility (Ma et al., 2025; Wang D, et al., 2026). HBB-based methods are simple and computationally efficient, but they usually infer picking points from fruit position or morphology, and their localization accuracy is limited because fruit orientation and slender stem structures are not explicitly represented (Huang et al., 2025; Tufail et al., 2026; Zhao et al., 2025). Segmentation-based methods can provide fruit masks, contours, or shape information for localization, but they mainly focus on the fruit body and may not fully model the corresponding stem structure, especially under occlusion (Tamrakar et al., 2025; Hu et al., 2022). Fruit OBB-based methods introduce orientation information into object representation and can better describe inclined fruits than HBB-based methods (Dong et al., 2023; Fu et al., 2025). Nevertheless, fruit-only OBB detection still relies primarily on fruit geometry and does not explicitly determine the true correspondence between fruits and stems, which limits the reliability of picking point localization in stem-cutting harvesting tasks (Dong et al., 2023).

Compared with fruit-only detection, simultaneous OBB-based detection of fruits and stems, together with explicit modeling of their geometric relationship, has greater potential to improve picking point localization accuracy. However, this approach still faces several challenges in practical applications. First, fruits and stems differ greatly in scale, and stems are slender and easily confused with the background. Second, severe occlusion between targets often leads to missed detections of small or slender targets. Third, the correspondence between fruits and stems is difficult to determine, which increases the uncertainty of picking point localization. Therefore, it is necessary to develop a vision-based method that can jointly detect fruits and stems and localize picking points based on the geometric relationship between their OBBs (Munir et al., 2026; Ye et al., 2025; Yu et al., 2020; Zhang et al., 2025).

Owing to their favorable balance between performance, speed, and compact architecture, YOLO-based models have been widely adopted in agricultural object detection tasks (Badgujar et al., 2024; Cong and Phuong, 2025). YOLOv11-OBB (You Only Look Once version 11-oriented bounding box) introduces orientation information into object detection by predicting oriented bounding boxes, making it suitable for the localization of direction-sensitive targets. However, when directly applied to fruit-stem detection in ridge-cultivated strawberry environments, it still has limited capability in detecting small targets and representing slender structural features, making it difficult to satisfy the perception requirements of complex field conditions. This study presents the FSGE-OBB model, an improved YOLOv11n-OBB-based model for fruit-stem oriented detection in ridge-cultivated strawberry fields, and further develops a fruit-stem association and picking point localization method. Key contributions of this study are:

1. An oriented bounding box dataset containing ripe fruit, half-ripe fruit, unripe fruit, and stem targets was constructed under ridge-cultivated strawberry field conditions, providing data support for fruit-stem oriented detection in complex harvesting environments.
2. An improved oriented object detection model, termed the FSGE-OBB model, was developed to enhance cross-scale feature interaction, directional representation of slender stems, and high-resolution detail recovery while maintaining a lightweight architecture.
3. A geometry-constrained fruit-stem association method was established by modeling the spatial relationship between ripe-fruit OBBs and stem OBBs, providing reliable fruit–stem correspondence for picking point localization.
4. A picking point localization method was developed for different detection scenarios, and its effectiveness was validated through preliminary three-dimensional localization experiments and simulated harvesting experiments on a strawberry-harvesting robot platform.

## 2 Materials and methods

### 2.1 Dataset construction and annotation

**Figure 1:**
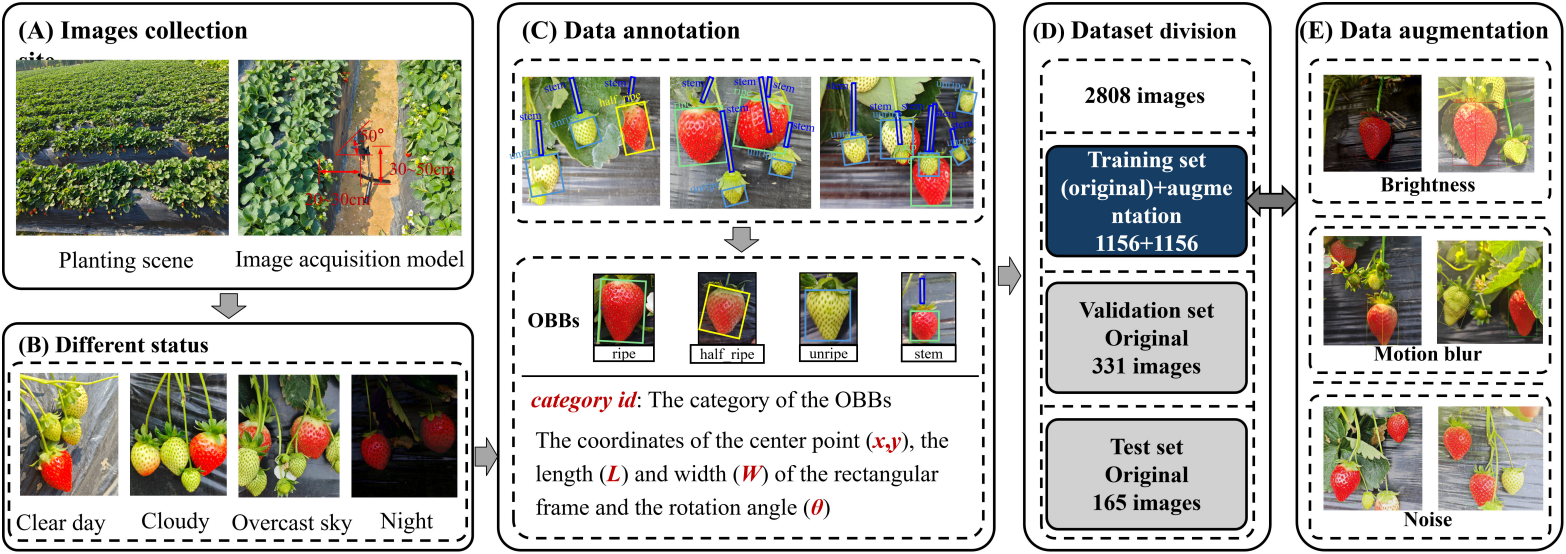
Dataset construction procedure. **(A)** Image acquisition scenarios and methods; **(B)** Different weather conditions during image acquisition; **(C)** Dataset annotation; **(D)** Dataset division; **(E)** Image data augmentation.

In this study, strawberry images were collected at Kaixin Strawberry Farm in Dongguan, Guangdong Province, China (22.9481° N, 113.7377° E). The farm adopts a ridge-cultivation mode. Images were captured using a smartphone (Redmi K70 Pro). During image acquisition, the distance between the camera and the strawberry ridge was maintained at 20–30 cm, the shooting height was 30–50 cm, and the angle between the camera and the horizontal plane was approximately 50°. Images were collected from January 27 to January 29, 2026, under different weather conditions, including sunny, cloudy, and overcast conditions, as well as under different occlusion levels. The acquired smartphone RGB images were used to construct the primary strawberry fruit-stem detection dataset for model training, validation, and testing. 1,760 strawberry images, each measuring 1920 × 1280 pixels, were collected, and **Figure 1** presents the full procedure used to construct the dataset.

After image screening, 1,652 valid images were retained. These images were initially randomly divided into training, validation, and test sets at a ratio of 70%, 20%, and 10%, corresponding to 1,156, 331, and 165 images, respectively. The resulting subsets were then reviewed to ensure an approximately balanced distribution of maturity stages, lighting/weather conditions, and occlusion levels. Minor adjustments were made when necessary to avoid severe imbalance among the subsets. To improve data diversity and model generalization, brightness perturbation, motion blur, and random noise injection were applied only to the training set. As a result, an additional 1,156 augmented images were generated, increasing the total number of training images from 1,156 to 2,312. No data augmentation was applied to the validation or test sets.

The open-source software X-AnyLabeling was employed to annotate the images with OBBs. Considering the significant differences in color, texture, and morphology among strawberries at different maturity stages, as well as the relatively high inter-class similarity between half-ripe and ripe fruits, the detection targets were divided into four categories: ripe fruit, half-ripe fruit, unripe fruit, and stem. The annotated data were then organized in a YOLO-compatible format and used for model training, validation, and testing.

### 2.2 Construction of the FSGE-OBB model

YOLOv11-OBB is a branch of the YOLOv11 family designed for oriented object detection. By introducing an angle parameter into conventional horizontal bounding boxes (HBBs), this model can represent arbitrarily oriented objects more compactly (Jun et al., 2025). In addition, it uses rotated non-maximum suppression (R-NMS) for candidate filtering, making it suitable for direction-sensitive detection tasks. The network mainly consists of a backbone, a neck, and a detection head, and incorporates modules such as C3k2, SPPF, and C2PSA to improve feature extraction and multi-level feature fusion.

**Figure 2:**
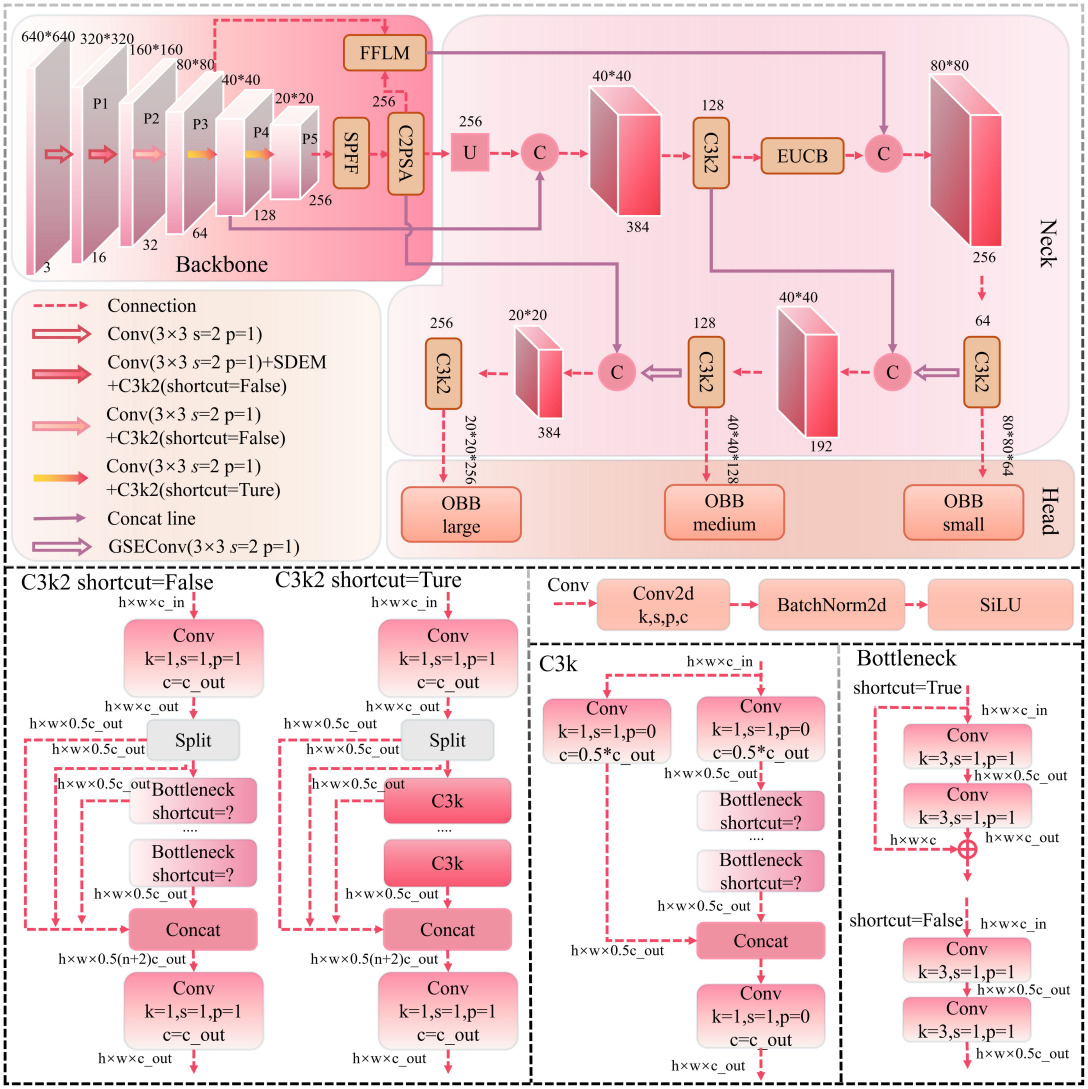
Framework of the proposed FSGE-OBB model.

YOLOv11n-OBB was selected as the baseline model because it provides a lightweight and modular OBB detection framework with a favorable balance between detection accuracy, model size, and computational cost, which is suitable for resource-constrained strawberry harvesting robots. However, when directly applied to ridge-cultivated strawberry-picking scenarios, YOLOv11n-OBB still suffers from insufficient detection accuracy for slender stems, missed detections of small targets, and false positives in complex backgrounds. To address these limitations, this study takes YOLOv11n-OBB as the baseline model and develops an improved model named FSGE-OBB for fruit–stem oriented object detection. The overall architecture of the proposed FSGE-OBB model is shown in **Figure 2**.

The architectural improvements were designed according to the visual characteristics of strawberry fruits and stems, aiming to improve the representation of small fruits and thin stems. First, FFLM was introduced to strengthen cross-layer fusion of low-level spatial features with high-level semantic cues, enhancing the representation of small fruits and slender stems. Second, SDEM was designed to enhance directional features of stems through asymmetric convolution branches and spatial weighting. Third, GSEConv was employed in the neck to reduce model complexity while preserving feature representation capability. Finally, EUCB was introduced into the high-resolution upsampling path to improve detail recovery and structural continuity for small and slender targets.

#### 2.2.1 Feature fusion lite module

**Figure 3:**
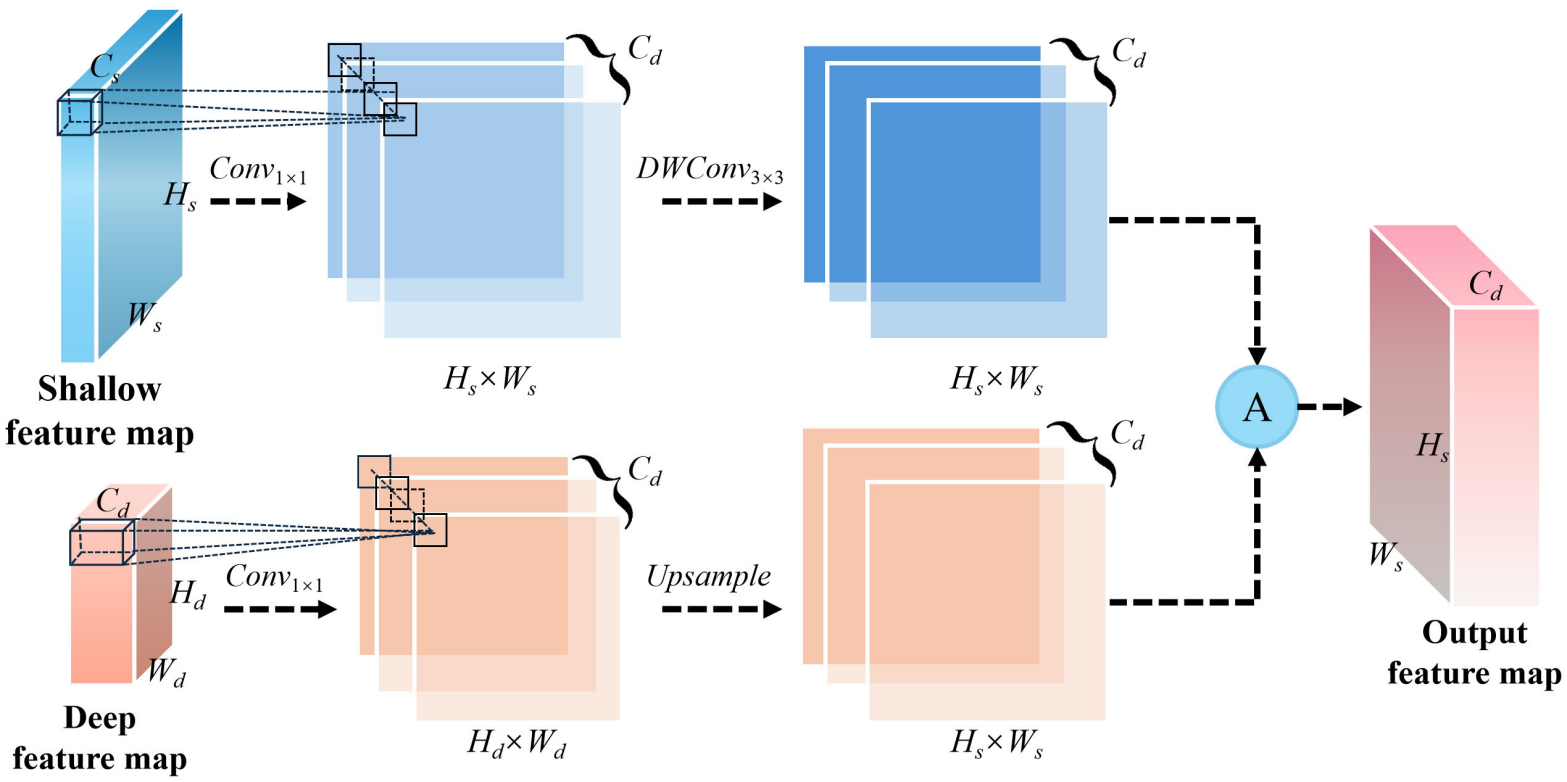
|Structure of the FFLM.

To improve the representation of small fruits and slender stems under complex background interference, a lightweight feature fusion module, termed FFLM, was introduced (**Figure 3**). Specifically, FFLM integrates high-resolution spatial details from the shallow feature map P3 with high-level semantic information from a deep feature map derived from the backbone output P5 after SPPF and C2PSA processing. Shallow features capture fine-grained structural information such as object edges and slender patterns, whereas deep features provide stronger semantic abstraction and robustness to background noise (Xu et al., 2026; Zhang et al., 2026). Consequently, cross-layer fusion between P3 and the deep feature map enhances multi-scale feature representation for accurate fruit and stem detection.

FFLM consists of a shallow branch and a deep branch. In the shallow branch, the input feature first passes through a 1×1 convolution for channel mapping, followed by a 3×3 depthwise convolution to extract local spatial information with low computational cost. In the deep branch, the input feature is first mapped by a 1×1 convolution and then upsampled to the same spatial resolution as the shallow feature. The shallow-detail feature and deep semantic feature are then fused by element-wise addition, and a final 1×1 convolution is used to integrate and remap the fused feature. Compared with the layer-by-layer information propagation scheme in conventional FPN (Feature Pyramid Networks)/PAN (Path Aggregation Networks) structures, FFLM directly establishes cross-layer interaction between shallow and deep features, shortens the information path, and improves multi-scale feature representation.

#### 2.2.2 Stem direction enhancement module

**Figure 4:**
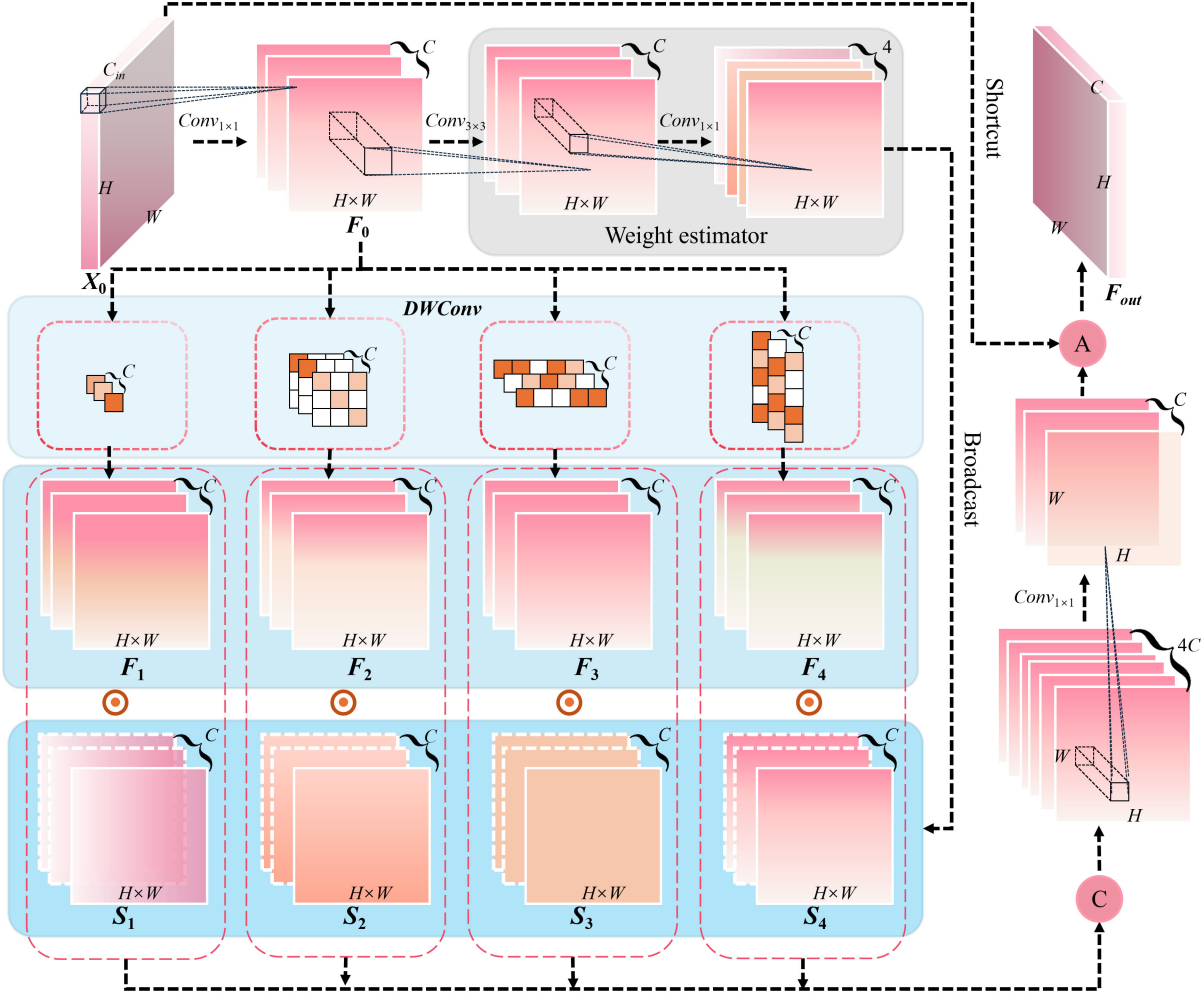
Structure of the SDEM.

Strawberry stems are slender, elongated, and direction-sensitive targets, and they usually occupy only a small number of pixels in the image. Conventional square-kernel convolutions mainly capture isotropic local patterns and may be insufficient for explicitly representing elongated horizontal or vertical stem structures. Although larger standard convolutions or deformable convolutions can increase the receptive field, they also introduce additional parameters and computational cost, which is unfavorable for lightweight robotic deployment. Therefore, SDEM was designed to enhance directional responses using asymmetric depthwise convolution branches, while a lightweight spatial weighting mechanism was used to adaptively emphasize informative directional features under different occlusion and orientation conditions. The structural diagram of SDEM is shown in **Figure 4**.

Given an input feature map $X_{0}\in R^{B\times C_{in}\times H\times W}$, a 1×1 convolution is first used for channel mapping and feature integration to obtain $F_{0}\in R^{B\times C\times H\times W}$. In this study, the input and output channels are kept the same, *C_in_*= *C*. Then, *F*_0_ is fed into four depthwise convolution (DWConv) branches in parallel, with kernel sizes of 1×1, 3×3, 1×5, and 5×1, respectively, to extract structural information under different receptive fields and directions, yielding four feature maps $F_{i}\in R^{B\times C\times H\times W}\left(i=1,2,3,4\right)$. Specifically, the 1×1 branch preserves fine details and channel information, the 3×3 branch extracts conventional local texture information, the 1×5 branch emphasizes horizontal slender structures, and the 5×1 branch emphasizes vertical slender structures.

Meanwhile, *F*_0_ is fed into a lightweight spatial weight estimator composed of a 3×3 convolution for channel reduction, a 1×1 convolution for mapping, and a sigmoid activation function, producing branch-wise spatial weights. These weights are used to perform pixel-wise gating on the corresponding branch features *F_i_*, resulting in enhanced features *S_i_*. The enhanced features are subsequently merged along the channel dimension, and a 1×1 convolution is applied to fuse the information and reduce the channel dimension. Finally, a residual connection is employed to generate the output feature *F_out_*. By explicitly modeling directional information through asymmetric convolutions and adaptively modulating branch responses with spatial weights, SDEM improves the model’s ability to perceive slender targets such as stems with low computational overhead.

#### 2.2.3 GSEConv module

**Figure 5:**
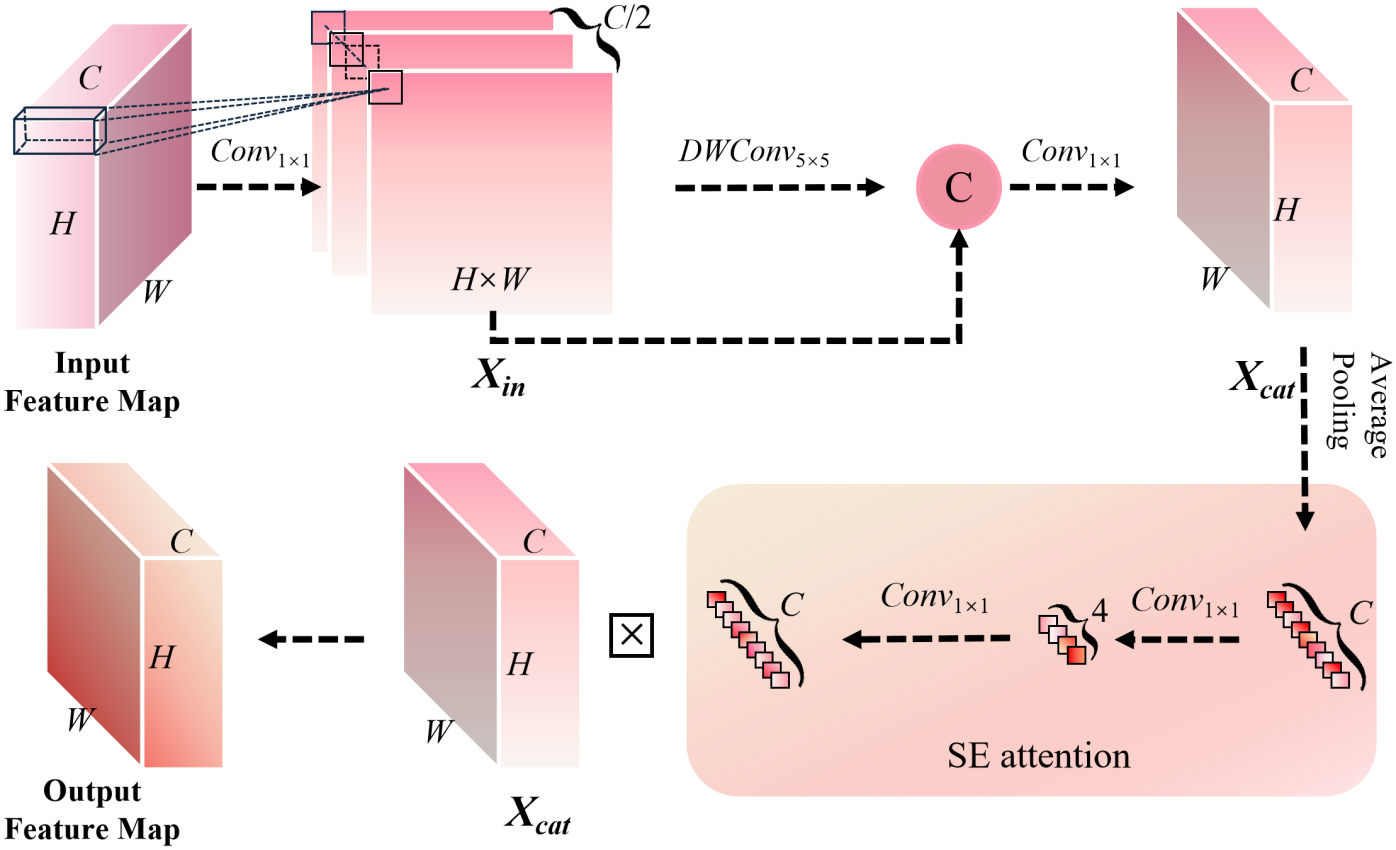
Structure of the GSEConv module.

In the proposed FSGE-OBB model, the neck is responsible for multi-scale feature fusion and therefore contributes significantly to the overall computational cost. While standard convolutions in the neck can enhance representation capability, they also substantially increase model size and GFLOPs. Conversely, overly lightweight designs (e.g., depthwise convolutions (DWConv) and GSConv) may reduce feature discrimination, particularly for challenging targets such as small fruits and slender stems. To address this trade-off, GSEConv is adopted as a balanced solution between efficiency and representational power (**Figure 5**). By integrating grouped spatial feature extraction with SE-based channel recalibration, GSEConv effectively reduces redundant computations while preserving informative feature responses, making it well-suited for resource-constrained strawberry harvesting robots (Zhang et al., 2026, Zhang and Zhang, 2026).

GSEConv consists of a main branch and an auxiliary branch. In the main branch, a 1×1 convolution is first applied for channel mapping and feature transformation, producing the feature map *X_in_*. Subsequently, *X_in_* is fed into the auxiliary branch, where a 5×5 DWConv is employed to expand the local receptive field and enrich spatial contextual information at a low computational cost, yielding an enhanced feature. The enhanced feature is then concatenated with *X_in_* along the channel dimension to form the fused feature *X_cat_*.

To further enhance informative features and suppress redundant responses, the SE (Squeeze-and-Excitation) module adaptively modulates feature responses across channels (Mahdiani, 2026). Specifically, global average pooling is first applied to extract channel-wise statistics, followed by two 1×1 convolution layers to generate the channel weight vector *w*. Finally, the fused feature *X_cat_* is reweighted by *w* to produce the output feature.

This module can replace part of the conventional convolution structure in the neck, enabling a lightweight design while maintaining strong feature representation capability.

#### 2.2.4 Efficient upsampling convolution block

**Figure 6:**
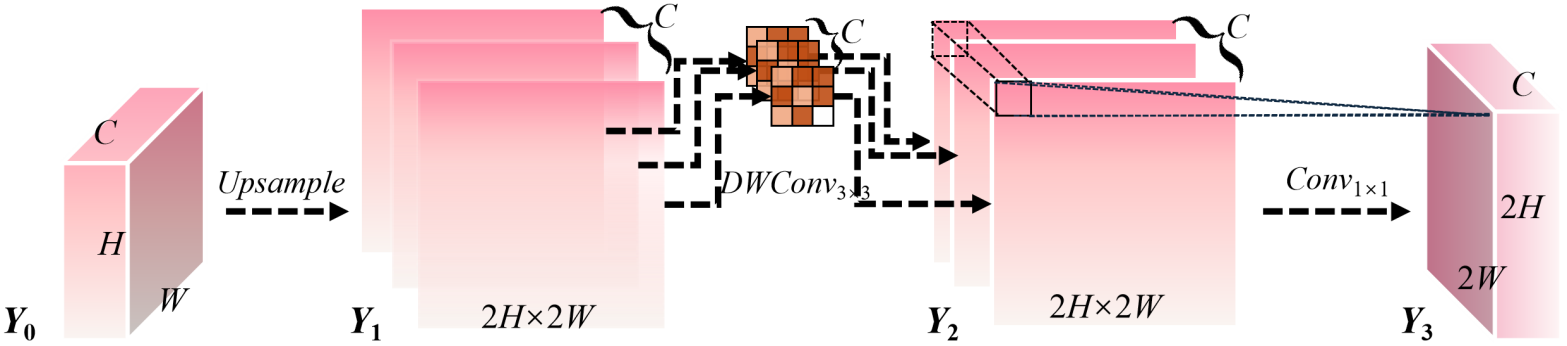
Structure of the EUCB.

In the feature pyramid, high-resolution feature maps are particularly important for detecting small fruits and thin stems. However, simple nearest-neighbor or bilinear upsampling only restores spatial resolution and does not further refine local structural details. Transposed convolution can learn upsampling parameters but may introduce additional computational cost and checkerboard artifacts. EUCB was therefore adopted to combine nearest-neighbor upsampling with depthwise convolution and pointwise convolution. The structure of EUCB is shown in **Figure 6**. This design enables local detail refinement after resolution recovery while keeping the computational overhead low, which is beneficial for improving the boundary continuity and structural representation of small and slender targets.

Assume that the input feature is *Y*_0_. First, nearest-neighbor interpolation is used to double the spatial resolution, generating the upsampled feature *Y*_1_. Then, a 3×3 DWConv is applied to *Y*_1_ to model local structures and alleviate the roughness and detail loss caused by direct interpolation, generating the locally enhanced feature map *Y*_2_. Finally, a 1×1 pointwise convolution is used to linearly combine and remap channel information, producing the enhanced output feature *Y*_3_, as described in Equation:


$$Y_{3}=PWConv_{1\times 1}\left(DWConv_{3\times 3}\left(UP\left(Y_{0}\right)\right)\right)$$

where *Y*_0_ is the input feature; *Y_3_* is the output enhanced feature map; $DWConv_{3\times 3}\left(\cdot \right)$ denotes a 3×3 depthwise convolution; $PWConv_{1\times 1}\left(\cdot \right)$ denotes a 1×1 pointwise convolution; *Up*(·) denotes nearest-neighbor upsampling.

In this study, EUCB was inserted into the upsampling path from P4 to P3 to improve the representation of small fruits and slender stems in high-resolution feature maps while preserving the semantic information of deeper layers.

### 2.3 Fruit-stem association and picking point localization

#### 2.3.1 Fruit-stem association method

**Figure 7:**
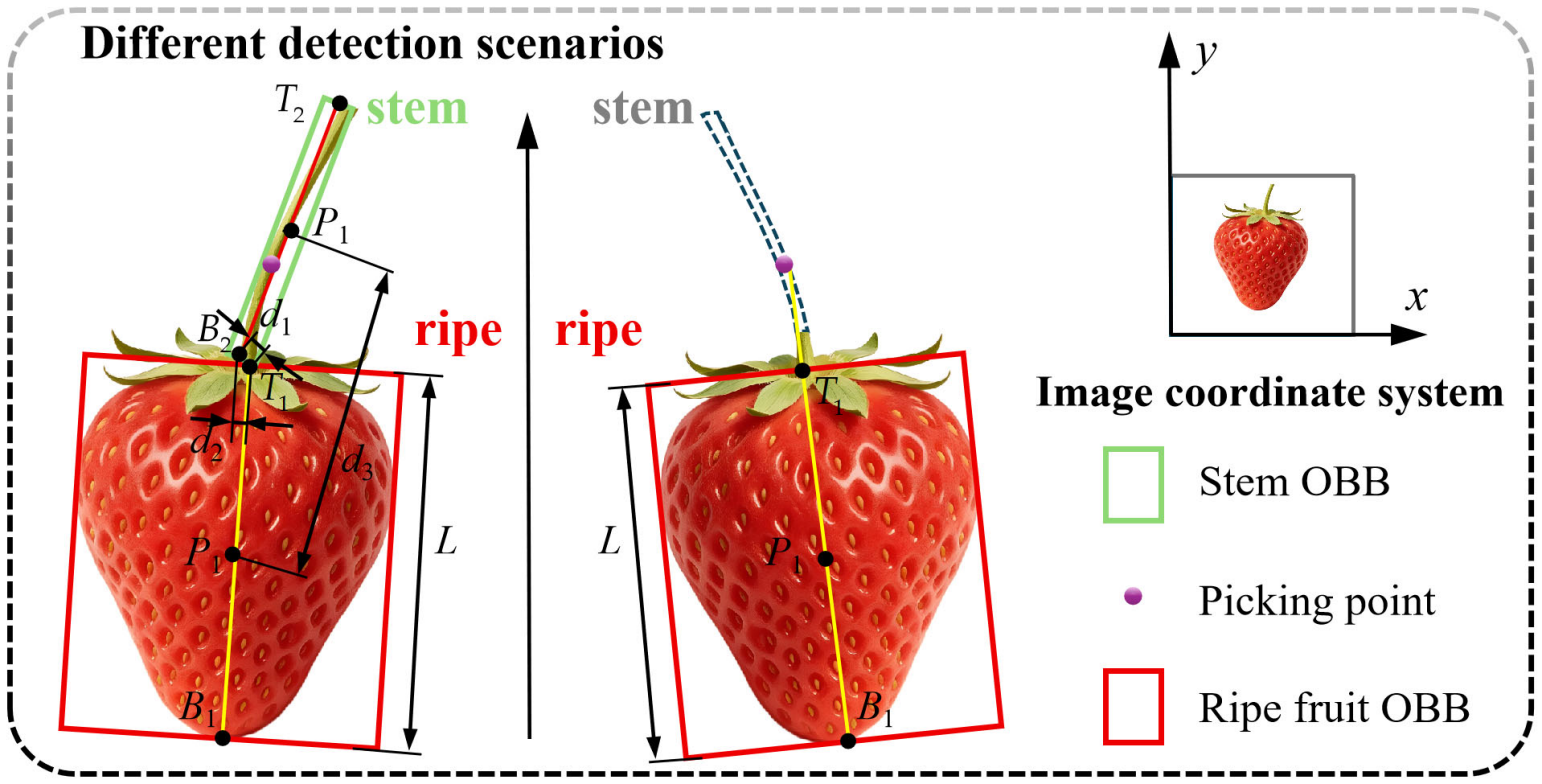
Fruit-stem geometric relationship based on OBBs.

After obtaining the oriented recognition outputs of ripe fruits and stems, the geometric information of the OBBs required for picking point localization was extracted in the image coordinate system. Based on the OBB parameters, the center points, major-axis directions, and the intersection points between the center lines and box boundaries of ripe fruits and stems were determined, as shown in **Figure 7**. Specifically, *B*_1_ denotes the lower intersection point between the fruit OBB boundary and its center line, referred to as the fruit lower endpoint; *T*_1_ denotes the corresponding upper intersection point, referred to as the fruit upper endpoint; *T*_1_*B*_1_ denotes the center-line segment of the fruit OBB, hereafter referred to as the fruit center-line segment; *P*_1_ denotes the center point of the fruit OBB, hereafter referred to as the fruit center point. Similarly, *B*_2_, *T*_2_, *T*_2_*B*_2_, and *P*_2_ denote the lower endpoint, upper endpoint, center-line segment, and center point of the stem OBB, respectively.

Because multiple ripe fruits and stems may be detected in a single image, it is necessary to determine whether a ripe fruit and a stem belong to the same strawberry before localizing the picking point. Therefore, a fruit-stem association method was developed based on the spatial geometric relationship between OBBs. Five geometric constraints were defined from the perspectives of local connection, global proximity, directional consistency, and connection-position rationality. A ripe fruit and a stem are regarded as associated only when all five constraints are satisfied simultaneously.

##### 2.3.1.1 Endpoint distance constraint

The stem is usually connected near the top of the strawberry. Therefore, the Euclidean distance between the stem lower endpoint *B*_2_ and the fruit upper endpoint *T*_1_ is constrained to determine whether the stem connection point is close to the fruit top.


$$d_{1}\leq \alpha_{1}L$$

where *d*_1_ is the Euclidean distance between points *T*_1_ and *B*_2_ (pixel); *α*_1_ is the endpoint distance coefficient; *L* is the major-axis length of the fruit OBB (pixel).

##### 2.3.1.2 Point-to-line distance constraint

Considering only the distance between *B*_2_ and *T*_1_ may still result in cases where the stem is located on the side of the fruit while remaining close to the fruit top. Therefore, the perpendicular distance from *B*_2_ to the fruit center-line segment *T*_1_*B*_1_ is further constrained to measure the lateral offset of the stem connection point relative to the fruit axis.


$$d_{2}\leq \alpha_{2}L$$

where *d*_2_ is the perpendicular distance from point *B*_2_ to line segment *T*_1_*B*_1_ (pixel); *α*_2_ is the point-to-line distance coefficient.

##### 2.3.1.3 Center point distance constraint

To further restrict candidate matching relationships at the global scale, the Euclidean distance between the stem center point *P*_2_ and the fruit center point *P*_1_ is constrained. This constraint is relatively loose and is mainly used to exclude stems that are obviously far from the fruit.


$$d_{3}\leq \alpha_{3}L$$

where *d*_3_ is the distance between points *P*_2_ and *P*_1_ (pixel); *α*_3_ is the center-point distance coefficient.

##### 2.3.1.4 Direction consistency constraint

To maintain a reasonable geometric connection between the fruit and its stem, the absolute cosine similarity between the direction vectors of the fruit center-line segment and the stem center-line segment is required to be greater than a predefined threshold.


$$a=\;\frac{\left|{\vec{B_{1}T}}_{1}\;\cdot {\vec{B_{2}T}}_{2}\right|}{\left|{\vec{B_{1}T}}_{1}\right|\;\left|{\vec{B_{2}T}}_{2}\right|}\;\geq \;\alpha_{4}$$

where *a* is the absolute cosine similarity between the direction vectors of the *T*_1_*B*_1_ and *T*_2_*B*_2_; *α*_4_ is the direction threshold.

##### 2.3.1.5 Projection-ratio constraint

Distance and direction constraints alone may still allow mismatches in which the stem connection point is projected to an unreasonable position along the fruit major axis. Therefore, the projection ratio *t_p_* of the stem lower endpoint *B*_2_ along the fruit center-line segment is introduced. Specifically, *t_p_* is used to describe the relative position of *B*_2_ with respect to the fruit lower endpoint *B*_1_ and fruit upper endpoint *T*_1_. When *t_p_* is close to 1, the stem lower endpoint is located near the fruit upper endpoint. Values smaller than 1 indicate that the projected point lies inside the fruit region, whereas values greater than 1 indicate that the stem endpoint is located above the fruit top along the extension of the fruit major axis. Since the actual stem is typically connected near the fruit top, constraining the range of *t_p_* can effectively avoid unreasonable mismatches.


$$\alpha_{5}\;\leq \;t_{p}\;=\;\frac{\vec{B_{1}B_{2}}\;\cdot \;\vec{B_{1}T_{1}}}{L^{2}}\;\leq \;\alpha_{6}$$

where *t_p_* is the projection ratio of the stem lower endpoint along the fruit center-line segment direction; *α*_5_ is the lower-bound projection coefficient; *α*_6_ is the upper-bound projection coefficient.

When a fruit and a stem satisfy the predefined association equations, they are considered a valid pair. To evaluate the performance of the proposed fruit-stem association method, the association success rate is adopted as the evaluation metric. The association success rate *Q* is defined as:


$$Q=\frac{N_{1}}{N_{0}}\times 100\%$$

where *Q* is the fruit-stem association success rate (%); *N*_0_ is the number of ripe fruits with actual corresponding stems; *N*_1_ is the number of ripe fruits correctly associated with their corresponding stems.

The five geometric constraints used in the proposed fruit-stem association method are controlled by six data-calibrated coefficients, namely *α*_1_-*α*_6_. To estimate these parameters under varying imaging viewpoints, a calibration subset of 500 unaugmented smartphone images was selected from the training set. The subset included overhead, oblique, and low-angle views and was used exclusively for parameter calibration, without participation in model evaluation.

Based on the OBB detection results obtained by the FSGE-OBB model, ripe fruits and their corresponding stems were manually checked. Only valid fruit-stem pairs with clear correspondence were retained for parameter statistics. For each valid pair, the normalized endpoint distance (r_1_), normalized point-to-line distance (r_2_), normalized center distance (r_3_), absolute cosine similarity (*a*), and projection ratio (t_p_) were calculated as follows:


$$r_{1}=d_{1}/L$$

$$r_{2}=d_{2}/L$$

$$r_{3}=d_{3}/L$$

where r_1_, r_2_, and r_3_ are the normalized endpoint distance, point-to-line distance, and center distance, respectively.

The statistical ranges of these geometric quantities were as follows: r_1_ in [0.007, 0.543], r_2_ in [0.007, 0.539], r_3_ in [0.676, 1.622], *a* in [0.7054, 1.000], and t_p_ in [1.0051, 1.547]. These results indicate that the geometric relationship between fruits and stems varies noticeably under different imaging viewpoints, especially when overhead and low-angle images are included.

To reduce the influence of a small number of abnormal samples while retaining most valid fruit–stem pairs, a percentile-based parameter selection strategy was adopted. For the upper-bound constraints, including r_1_, r_2_, r_3_, and the upper bound of t_p_, the 95th percentile was used as the threshold. For the lower-bound constraints, including *a* and the lower bound of t_p_, the 5th percentile was used as the threshold. Accordingly, the final parameters were determined as follows: *α*_1_ = 0.53, *α*_2_ = 0.52, *α*_3_ = 1.59, *α*_4_ = 0.72, *α*_5_ = 1.006, *α*_6_ = 1.51.

Finally, a single-factor sensitivity analysis was conducted on the test set, which contained 165 strawberry images, to evaluate the robustness of the association parameters. The test set was not used for model training or parameter calibration. During this *post-hoc* analysis, each parameter was varied individually with a step size of 0.05 while keeping the remaining parameters fixed at the calibrated values, and the fruit-stem association success rate was used as the performance metric.

#### 2.3.2 Picking point localization method

After the fruit-stem association was determined, two picking point localization strategies were designed for different OBB detection scenarios based on the completeness of the detection results and the practical requirements of harvesting.

1. When both a ripe fruit and its corresponding stem are detected, the picking point is set along the direction of the stem center-line segment at a position 18 mm above the stem lower endpoint, so as to better satisfy the requirements of actual stem-cutting operations.

2. When only the ripe fruit is detected and its corresponding stem is not detected, the picking point is set along the direction of the fruit center-line segment at a position 20 mm above the fruit upper endpoint, ensuring that the cutting position is as close as possible to the fruit-stem connection region.

The 18 mm offset was selected to ensure that the cutting point remained above the fruit-stem junction while avoiding damage to the fruit surface. The 20 mm offset in the fruit-only case was slightly larger to compensate for the uncertainty caused by missing stem detection.

In strawberry-harvesting tasks, depth cameras are commonly used to obtain the spatial position of picking points. Therefore, this study further investigates picking point localization in the depth camera coordinate system. The depth camera acquires both RGB and depth images of strawberries, and coordinate alignment between the depth image and the RGB image is first performed. Subsequently, the FSGE-OBB model is applied to perform oriented object detection of fruits and stems, and the pixel-level parameters of their OBBs are obtained. According to the proposed picking point localization strategies, the two-dimensional (2D) pixel coordinates of the picking point are first calculated in the RGB image coordinate system and then converted into three-dimensional (3D) coordinates in the depth camera coordinate system.

1. When both the ripe fruit and its corresponding stem are detected.

In this case, the center line of the stem OBB is obtained, and the pixel coordinate vectors of the upper and lower endpoints of the stem OBB in the image coordinate system are denoted as $\vec{T_{2}}={\left(x_{0},y_{0}\right)}^{T}$ and $\vec{B_{2}}={\left(x_{1},y_{1}\right)}^{T}$. The unit direction vector along the stem center-line segment from the stem lower endpoint to the stem upper endpoint is then calculated as follows:


$$\vec{v}=\left(v_{x},v_{y}\right)=\frac{\vec{B_{2}T_{2}}}{\left|\vec{B_{2}T_{2}}\right|}=\frac{(x_{0}-x_{1},y_{0}-y_{1}}{\sqrt{{\left(x_{0}-x_{1}\right)}^{2}+{\left(y_{0}-y_{1}\right)}^{2}}}$$

where $\vec{v}$ is the unit direction vector along the stem center-line segment.

The picking point is then offset upward by 18 mm from the lower endpoint along the stem center-line segment. Since 18 mm is a physical distance, it cannot be directly used as a pixel offset and must be converted into a pixel offset *t*_0_ using the depth value and camera intrinsic parameters.


$$t_{0}=\frac{d_{m0}}{Z_{B}\sqrt{{\left(\frac{v_{x}}{f_{x}}\right)}^{2}+{\left(\frac{v_{y}}{f_{y}}\right)}^{2}}}$$

where *t*_0_ is the pixel offset matched with 18 mm physical distance (pixel); *d_m_*_0_ is the physical offset distance (mm), set to 18 mm; *Z_B_*is the depth value at the *B*_2_ in the depth camera coordinate system (mm); *fx* and *fy* represent the focal lengths of the depth camera in the *x*- and *y-*directions.

Finally, the pixel coordinate vector of the picking point in this case can be expressed as:


$$\vec{P_{K}}={\vec{B}}_{2}+t_{0}\vec{v}$$

where $\vec{P_{K}}$ is the two-dimensional pixel coordinate vector of the picking point in the image coordinate system.

(2) Only the ripe fruit is detected.

In this case, the pixel coordinate vectors of the fruit upper endpoint and the fruit lower endpoint are obtained as $\vec{T_{1}}={\left(x_{2},y_{2}\right)}^{T}$ and $\vec{B_{1}}={\left(x_{3},y_{3}\right)}^{T}$. The unit direction vector along the fruit center-line segment from the fruit lower endpoint to the fruit upper endpoint is then calculated as follows:


$$\vec{u}=\left(u_{x},u_{y}\right)=\frac{\vec{B_{1}T_{1}}}{\left|\vec{B_{1}T_{1}}\right|}=\frac{\left(x_{2}-x_{3},y_{2}-y_{3}\right)}{\sqrt{{\left(x_{2}-x_{3}\right)}^{2}+{\left(y_{2}-y_{3}\right)}^{2}}}$$

where $\vec{u}$ is the unit direction vector along the fruit center-line segment.

The picking point is then offset upward by 20 mm from the fruit upper endpoint along the fruit center-line segment. By the same logic as above, this physical offset is converted to a pixel offset *t*_1_ in the image coordinate system.


$$t_{1}=\frac{d_{m1}}{Z_{T}\sqrt{{\left(\frac{u_{x}}{f_{x}}\right)}^{2}+{\left(\frac{u_{y}}{f_{y}}\right)}^{2}}}$$

where *t*_1_ is the pixel offset corresponding to a physical distance of 20 mm (pixel); *d_m_*_1_ is the physical offset distance (mm), set to 20 mm; *Z_T_* is the depth value at the *T*_1_ in the depth camera coordinate system (mm).

Finally, the pixel coordinate vector of the picking point in this case can be expressed as:


$$\vec{P_{k}}={\vec{T}}_{1}+t_{1}\vec{u}$$

Once the picking point’s pixel coordinates $\vec{P_{k}}={\left(u_{k},v_{k}\right)}^{T}$, are obtained, they are back-projected into three-dimensional camera coordinates using the aligned depth map and camera intrinsics via the pinhole camera model, thereby yielding the spatial position of the picking point. The transformation relationship is:


$$X_{k}=\left(u_{k}-c_{x}\right)\frac{Z_{k}}{f_{x}}$$

$$Y_{k}=\left(v_{k}-c_{y}\right)\frac{Z_{k}}{f_{y}}$$

$$Z_{k}=d\left(u_{k},v_{k}\right)$$

where *X_k_*, *Y_k_*, *Z_k_* are the 3D coordinates of the picking point in the depth camera coordinate system (mm); (*u_k_*, *v_k_*) are the pixel coordinates of the picking point in the image coordinate system; *d*(*u_k_*, *v_k_*) denotes the depth value of picking point in the depth camera coordinate system; *c_x_* and *c_y_* are the camera principal point coordinates.

Accordingly, the three-dimensional coordinates of the picking point in the depth camera coordinate system can be obtained as:


$$P_{k}={\left(X_{k},Y_{k},Z_{k}\right)}^{T}$$

**Figure 8:**
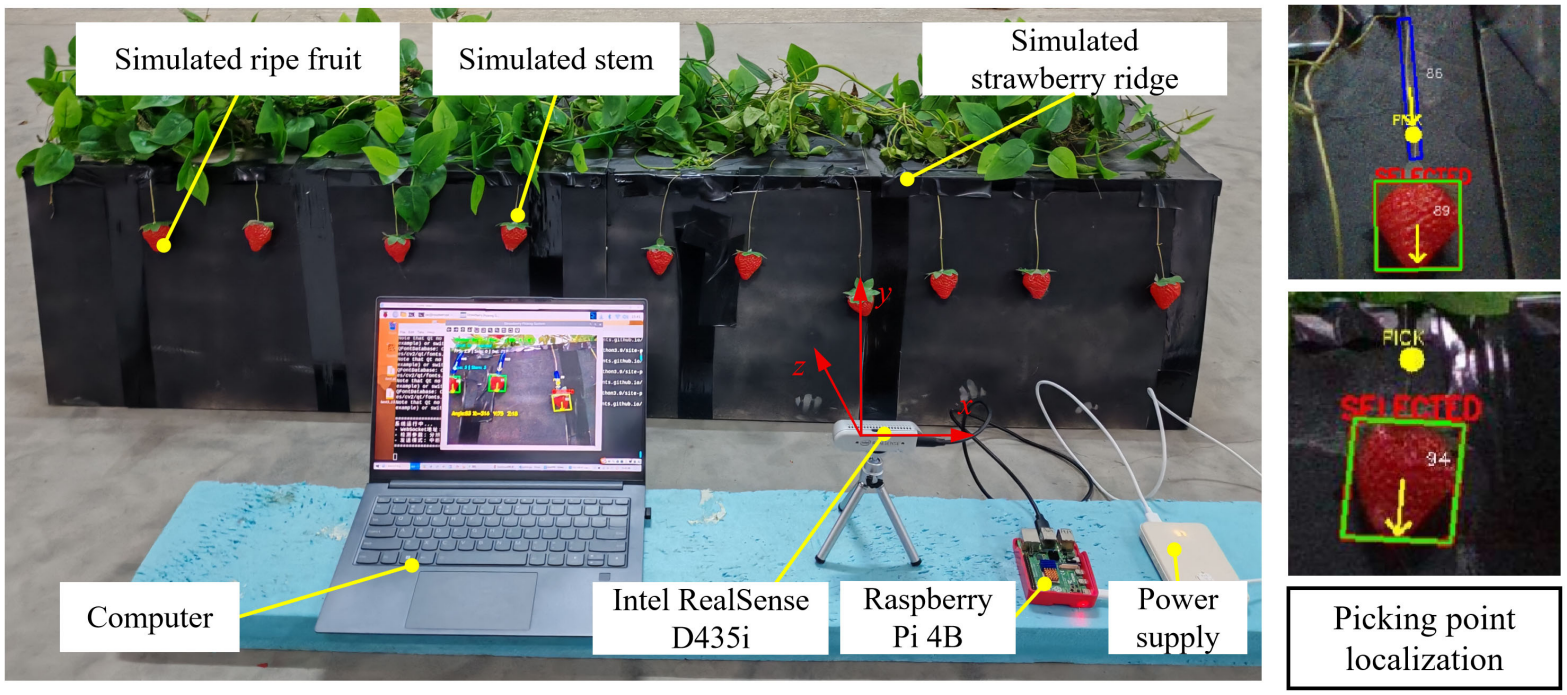
Ridge-cultivated strawberry harvesting simulation platform and picking point localization process.

In order to evaluate the effectiveness of the proposed picking point localization, a ridge-cultivated strawberry localization simulation platform was developed to perform three-dimensional localization experiments in the depth camera coordinate system. The platform consisted of a simulated strawberry ridge, an Intel RealSense D435i depth camera, a Raspberry Pi 4B, a host computer, and a power supply, as illustrated in **Figure 8**. The depth camera was used to acquire RGB and depth images of simulated strawberries and transmit the image data to the Raspberry Pi. The Raspberry Pi ran the FSGE-OBB model for fruit and stem detection, performed fruit-stem association using the proposed geometric constraint-based method, calculated the 2D pixel coordinates of the picking point according to the corresponding localization strategy, and then obtained the predicted 3D coordinates in the depth camera coordinate system through depth back-projection.

It should be noted that the RGB image dataset collected using the smartphone was employed to develop and validate the detection model, whereas the RealSense D435i depth camera was used only in the picking point localization experiment to obtain aligned RGB-D data.

Based on the above procedure, the predicted picking point coordinate in the depth camera coordinate system is defined as:


$$P_{rk}={\left(X_{rk},Y_{rk},Z_{rk}\right)}^{T}$$

where *P_rk_* is the predicted picking point coordinate in the depth camera coordinate system.

Meanwhile, the ground-truth picking point coordinate through manual calibration is denoted as:


$$P_{gk}={\left(X_{tk},Y_{tk},Z_{tk}\right)}^{T}$$

where *P_gk_* is the ground-truth picking point coordinate in the depth camera coordinate system.

For quantitative evaluation, the predicted picking point coordinates were compared to the ground-truth coordinates obtained through manual calibration. A total of 12 ripe fruits were tested, and the mean and maximum absolute localization errors along the *x-*, *y-*, and *z-*directions were recorded.

#### 2.3.3 Simulated harvesting experiment

**Figure 9:**
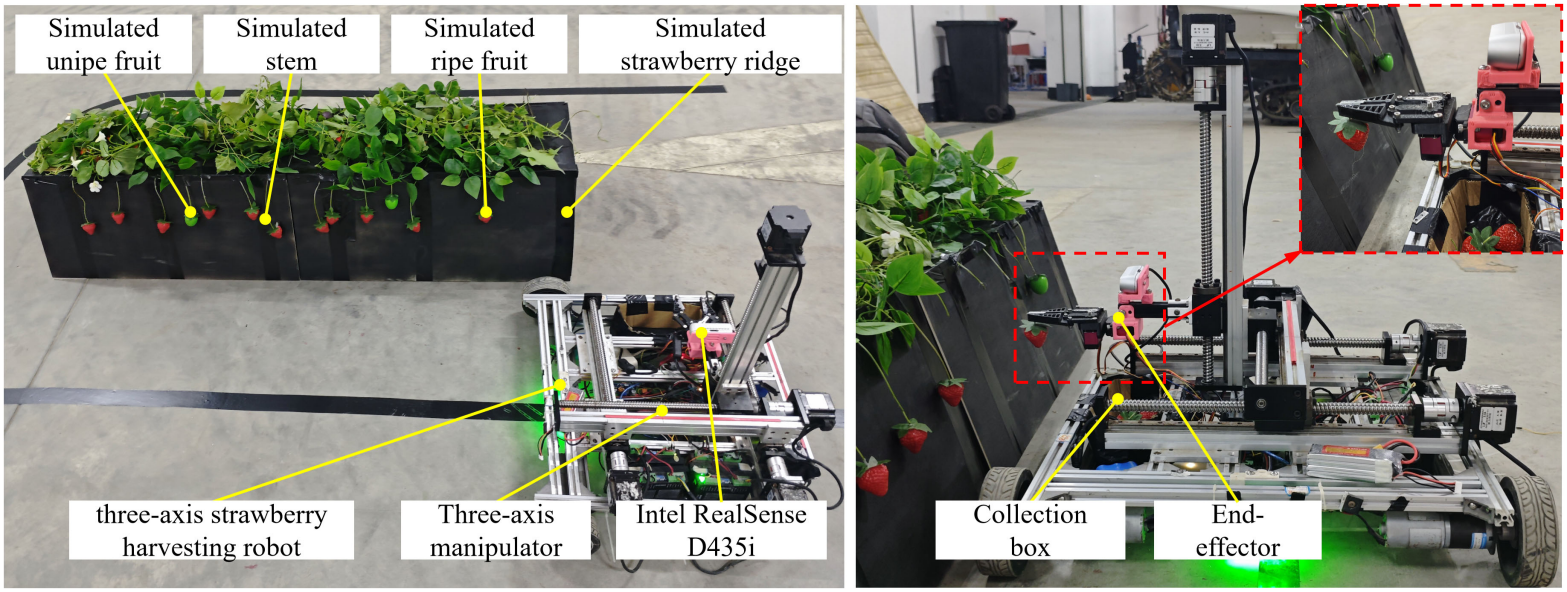
Three-axis strawberry harvesting robot and harvesting process.

To evaluate whether the FSGE-OBB model satisfies the visual perception requirements of a strawberry harvesting robot, and to verify the effectiveness of the proposed picking-point localization method, a simulated harvesting experiment was conducted using a self-developed three-axis strawberry harvesting robot, as shown in **Figure 9**.

The experimental platform consisted of a three-axis manipulator, an end-effector, an Intel RealSense D435i depth camera, and an NVIDIA Jetson Orin NX with 8 GB memory. The depth camera was used for RGB-D image acquisition, while the Jetson Orin NX served as the onboard computing platform for image processing, target detection, and picking-point localization. To enable accurate spatial positioning of strawberries in the robot base coordinate system, hand-eye calibration was further required to establish the transformation relationship between the camera and robot base frames.

The 3D picking point solved in Section 2.3.2 is defined under the depth camera coordinate system as $P_{k}={\left(X_{k},Y_{k},Z_{k}\right)}^{T}$. However, this coordinate cannot be directly used for manipulator motion control. Therefore, hand-eye calibration was performed to establish the rigid spatial relationship between the camera coordinate system and the robot base coordinate system. Seventeen calibration board observations from multiple robot poses were used to solve the hand-eye calibration problem. Through hand-eye calibration, the extrinsic transformation between the camera coordinate system and the robot base coordinate system was obtained, resulting in the following rigid transformation matrix ( $T_{B}^{C}$):


$$T_{B}^{C}=\left[\begin{array}{cc} R_{B}^{C} & t_{B}^{C}\\ 0 & 1 \end{array}\right]$$

Where $R_{B}^{C}\in {\text{R}}^{3\times 3}$ is the rotation matrix and $t_{B}^{C}\in {\text{R}}^{3\times 1}$ is the translation vector from the camera coordinate system to the robot base coordinate system. According to the calibration results, $R_{B}^{C}$ and $t_{B}^{C}$ were obtained as:


$$R_{B}^{C}=\left[\begin{array}{ccc} 0.9955346 & 0.0085195 & 0.0942543\\ -0.0073977 & 0.9998643 & -0.0128652\\ -0.0945492 & 0.0138292 & 0.9954627 \end{array}\right]$$

$$t_{B}^{C}=\left[\begin{array}{c} 35.4752324\\ 119.9982862\\ 15.8762963 \end{array}\right]$$

Using this calibrated transformation, the picking point was mapped from the camera coordinate system to the robot base coordinate system as follows:


$$P_{B}^{k}=R_{B}^{C}\cdot P_{k}+t_{B}^{C}$$

where $P_{B}^{k}={\left(X_{B},Y_{B},Z_{B}\right)}^{T}$ denotes the 3D coordinate of the picking point in the robot base coordinate system. This transformed coordinate $P_{B}^{k}$ was then used as the target input for inverse kinematics and robotic motion execution.

During the simulated harvesting process, the depth camera first acquired RGB-D images of the simulated strawberry scene. The images were then processed by the FSGE-OBB model deployed on the Jetson Orin NX to detect ripe strawberries and determine their picking points. After coordinate transformation, the picking point in the robot base coordinate system was transmitted to the three-axis manipulator. The manipulator moved the end-effector to the target position, performed stem cutting and grasping, and finally transported the fruit to the collection box.

The experiment was conducted on the simulated strawberry harvesting platform at South China Agricultural University. The simulated harvesting test was carried out three times, with 20 harvesting attempts per trial, resulting in a total of 60 harvesting cycles. For each trial, the picking point localization time for a single simulated fruit, the total harvesting time, and harvesting success or failure were recorded. Based on these data, the simulated harvesting success rate *Q_p_* was calculated.


$$Q_{p}=\frac{N_{2}}{N_{1}}\times 100\%$$

where *Q_p_* is the simulated harvesting success rate (%); *N*_1_ is the total number of simulated ripe fruits; *N*_2_ is the number of successfully harvested simulated ripe fruits.

The total harvesting time is defined as a complete closed-loop operational cycle, starting from RGB-D image acquisition and ending with the successful placement of the harvested strawberry into the collection box. This cycle includes visual inference, picking point localization, robot motion planning, manipulator trajectory execution, stem cutting and grasping, transportation, and return-to-home motion. Mobile chassis navigation or relocation time is excluded from the total harvesting time.

### 2.4 Evaluation metrics

Model performance was assessed using several metrics, including Precision (*P*), Recall (*R*), F1 score, mean average precision (mAP@0.5 and mAP@0.5:0.95), model size, number of parameters, and GFLOPs. All detection metrics were obtained on the smartphone-acquired test set. Detection performance was evaluated based on Precision, Recall, F1 score, and mAP, while model complexity and deployment potential were analyzed through model size, parameter count, and GFLOPs. All metrics were calculated on the test set to provide a comprehensive evaluation of the FSGE-OBB model in terms of both detection accuracy and deployment efficiency.


$$P=\frac{TP}{TP+FP}\times 100\%$$

$$R=\frac{TP}{TP+FN}\times 100\%$$

$$AP=\int_{0}^{1}p\left(R\right)dR$$

$$mAP=\frac{{\sum_{i=1}^{n}AP}_{i}}{n}$$

where *T_P_* represents the number of positive samples correctly predicted as positive; *F_P_* represents the number of negative samples incorrectly predicted as positive; *F_N_* represents the number of positive samples incorrectly predicted as negative; *AP* represents the area under the Precision-Recall curve; *mAP* represents the average *AP* over all categories.

## 3 Experimental results and analysis

### 3.1 Experimental setup

All experiments were performed on a Windows 11 workstation equipped with an NVIDIA RTX 4070 GPU with 12 GB of memory. The software environment consisted of Python 3.9, PyTorch 2.1.0, Anaconda 3, and CUDA 11.3. During training, the input resolution was set to 640×640, the batch size was set to 32, and the model was trained for 200 epochs. AdamW was adopted as the optimizer, with an initial learning rate of 0.001 and a weight decay of 0.0005. Other hyperparameters followed the default configuration of the training framework.

### 3.2 Ablation study

We sequentially incorporated FFLM, SDEM, GSEConv, and EUCB into the YOLOv11n-OBB baseline, and **Tables 1** and **2** summarize the ablation findings.

**Table 1:** Overall results of the ablation study.

Model					Global indicator							
YOLOv11n-OBB	FFLM	SDEM	GSEConv	EUCB	Precision (%)	Recall (%)	F1 score (%)	mAP@0.5 (%)	mAP@0.5:0.95 (%)	Model size (MB)	Parameters (M)	GFLOPs
✓					72.26	75.23	73.72	76.26	66.37	5.51	2.65	10.25
✓	✓				71.88	77.43	74.55	77.83	67.41	5.69	2.75	11.51
✓	✓	✓			75.23	73.45	74.33	78.18	66.95	5.73	2.76	12.24
✓	✓	✓	✓		70.65	76.56	73.49	77.62	66.77	5.56	2.67	12.07
✓	✓	✓	✓	✓	72.59	77.02	74.74	78.85	68.27	5.60	2.68	12.43

**Table 2:** Class-wise results of the ablation study.

Model					Ripe fruit		Half-ripe fruit		Unripe fruit		Stem	
YOLOv11n-OBB	FFLM	SDEM	GSEConv	EUCB	mAP@0.5 (%)	mAP@0.5:0.95 (%)	mAP@0.5 (%)	mAP@0.5:0.95 (%)	mAP@0.5 (%)	mAP@0.5:0.95 (%)	mAP@0.5 (%)	mAP@0.5:0.95 (%)
✓					86.33	81.61	77.64	75.79	79.45	67.52	61.63	40.54
✓	✓				86.19	81.89	80.40	75.35	80.81	69.70	63.91	42.68
✓	✓	✓			88.39	83.27	79.89	74.56	79.68	68.47	64.76	41.51
✓	✓	✓	✓		86.64	82.70	78.95	74.21	80.01	69.15	64.87	41.02
✓	✓	✓	✓	✓	88.97	83.12	77.77	75.68	83.20	71.42	65.47	42.86

For the baseline YOLOv11n-OBB, Precision, Recall, F1 score, mAP@0.5, and mAP@0.5:0.95 were 72.26%, 75.23%, 73.72%, 76.26%, and 66.37%, respectively. After introducing FFLM, Recall increased from 75.23% to 77.43%, while mAP@0.5 rose from 76.26% to 77.83%, and mAP@0.5:0.95 from 66.37% to 67.41%. These results suggest that cross-layer fusion between shallow spatial details and deep semantic information improves multi-scale feature representation. In the class-wise results, the mAP@0.5 of the stem category increased from 61.63% to 63.91%, indicating that FFLM is beneficial for detecting slender and small-scale targets.

After further introducing SDEM, Precision increased to 75.23% and mAP@0.5 increased to 78.18%. The class-wise results show that the mAP@0.5 values of ripe fruit and stem increased to 88.39% and 64.76%, respectively. This finding suggests that SDEM enhances the representation of direction-sensitive targets, especially ripe fruits and stems with evident orientation characteristics.

When GSEConv was added, the number of parameters decreased from 2.76 M to 2.67 M, the model size decreased from 5.73 MB to 5.56 MB, and GFLOPs decreased from 12.24 to 12.07. However, Precision, F1 score, mAP@0.5, and mAP@0.5:0.95 slightly decreased compared with those of the previous stage. This result indicates that GSEConv mainly contributes to model compactness and computational reduction, although it introduces a slight loss in feature representation.

After introducing EUCB, the model reached its highest detection performance, with Precision, Recall, F1 score, mAP@0.5, and mAP@0.5:0.95 attaining 72.59%, 77.02%, 74.74%, 78.85%, and 68.27%, respectively. Relative to the original baseline, mAP@0.5 and mAP@0.5:0.95 increased by 2.59 and 1.90 percentage points, while Recall and F1 score improved by 1.79 and 1.02 percentage points. Among all categories, the stem category showed the largest improvement, with mAP@0.5 increasing from 61.63% to 65.47%. This result indicates that the proposed model is particularly effective in improving the detection of difficult small and slender targets.

Overall, the sequential ablation results are consistent with the expected design objectives of the proposed modules. FFLM improves multi-scale feature fusion, SDEM enhances directional representation for stems, GSEConv reduces model complexity, and EUCB improves high-resolution detail recovery. By combining these modules, the FSGE-OBB model achieves a favorable balance between detection accuracy and compact design, especially for small fruits and slender stems in ridge-cultivated strawberry scenes.

### 3.3 Comparison experiments

To further evaluate the performance of the FSGE-OBB model, it was compared with several representative object detection models under the same dataset partition and training settings. The comparison results are shown in **Table 3**.

**Table 3:** Comparison of test results.

Model	Precision (%)	Recall (%)	F1 score (%)	mAP@0.5 (%)	mAP@0.5:0.95 (%)	Model size (MB)	Parameters (M)	GFLOPs
YOLOv6n-OBB	72.57	73.35	72.96	77.38	67.18	8.74	4.35	18.73
YOLOv8n-OBB	70.47	75.78	73.03	77.10	66.71	5.65	2.76	11.05
YOLOv10n-OBB	71.71	74.54	73.10	76.03	66.58	5.79	2.77	13.36
YOLOv11n-OBB	72.26	75.23	73.72	76.26	66.37	5.51	2.65	10.25
YOLOv12n-OBB	71.56	76.30	73.85	77.47	67.58	5.55	2.63	10.26
YOLOv26n-OBB	72.66	75.72	74.16	76.17	66.11	5.58	2.45	8.50
RT-DETR-ResNet50	72.16	77.59	74.78	76.40	65.38	47.18	25.42	91.12
FSGE-OBB	72.59	77.02	74.74	78.85	68.27	5.60	2.68	12.43

* The RT-DETR-ResNet50 model has replaced the detection head with an OBB head.

The FSGE-OBB model achieved mAP@0.5 and mAP@0.5:0.95 values of 78.85% and 68.27%, respectively, which were higher than those of the compared models. Its Recall reached 77.02%, indicating a stronger ability to detect strawberry fruits and stems with fewer missed detections. The F1 score of FSGE-OBB was 74.74%, which was close to that of RT-DETR-ResNet50 and higher than those of most lightweight YOLO-series models.

The FSGE-OBB model has a model size of 5.60 MB, 2.68 M parameters, and 12.43 GFLOPs. Compared with YOLO-based models of similar size, the FSGE-OBB model achieved higher mAP and Recall while maintaining a compact model size. Although RT-DETR-ResNet50 achieved a comparable F1 score, its model size, number of parameters, and computational cost were substantially higher, rendering it less viable for resource-constrained agricultural robotics.

These results suggest that the FSGE-OBB model achieves a favorable detection accuracy-compactness trade-off for strawberry fruit and stem detection in ridge-cultivated environments. However, its GFLOPs are higher than those of the YOLOv11n-OBB baseline, indicating that further optimization is still needed for embedded real-time deployment.

**Figure 10:**
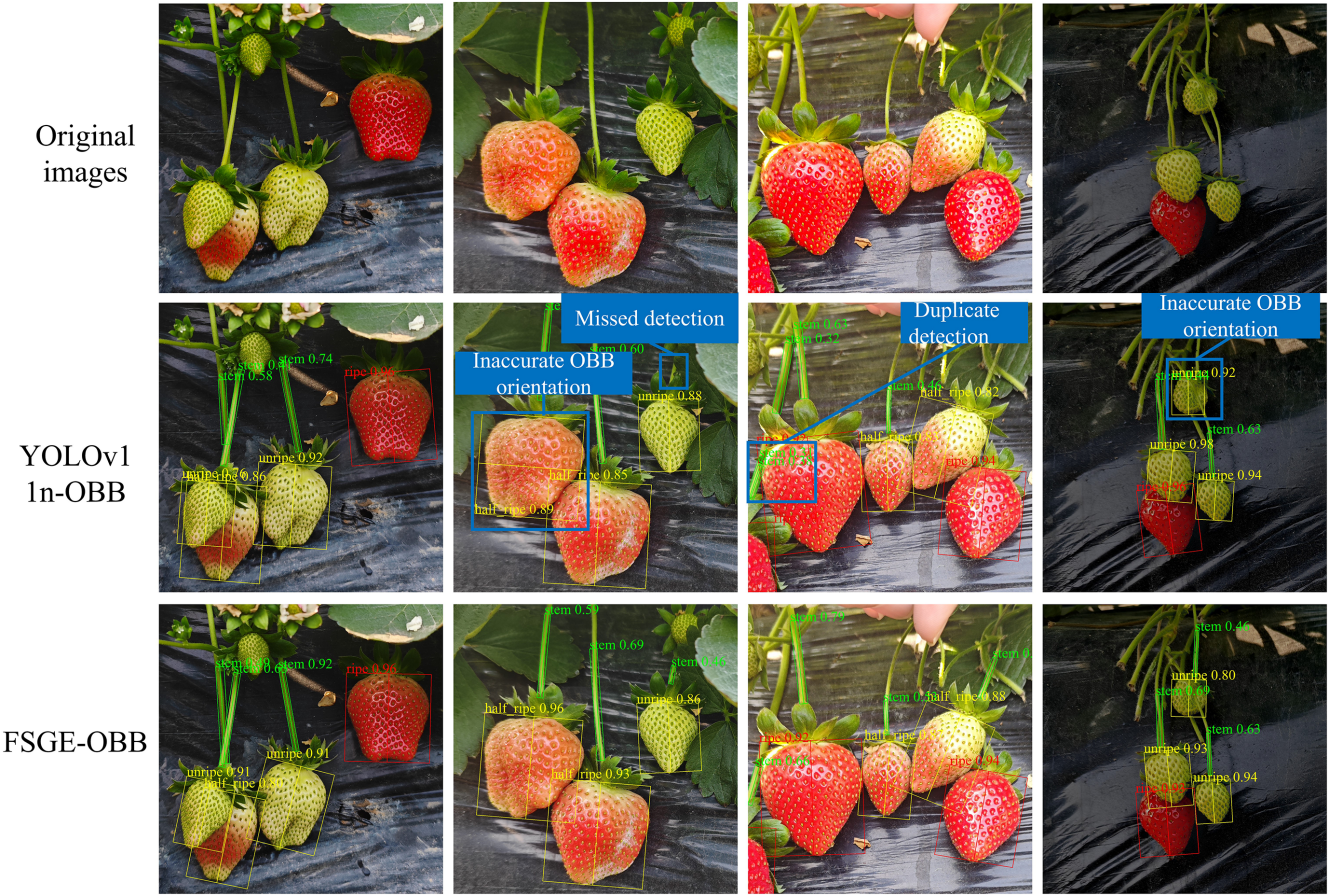
Comparison results between YOLOv11n-OBB and FSGE-OBB models.

To further compare the detection performance before and after model improvement, the FSGE-OBB model was visually compared with the baseline YOLOv11n-OBB (see **Figure 10**). The baseline model is more likely to produce missed detections and duplicate detections under insufficient illumination, mutual occlusion, and small object size. In particular, it shows limited capability in detecting slender stems and small fruits, and may generate inaccurate OBB orientation estimates for strawberry fruits. In contrast, the FSGE-OBB model produces more complete detection results for both fruits and stems. The improved model reduces missed detections of small targets and provides more stable OBB localization under occlusion and background interference. These visual results are consistent with the quantitative results in **Tables 1**–**3**.

### 3.4 Transferability of the proposed modules to YOLOv26n-OBB

To further evaluate the generalization capability of the proposed modules, additional experiments were conducted using YOLOv26n-OBB, a more recent lightweight oriented object detector. The results are summarized in **Tables 4** and **5**.

**Table 4:** Overall performance comparison of YOLOv26n-OBB with proposed modules.

Model					Global indicator							
YOLOv26n-OBB	FFLM	SDEM	GSEConv	EUCB	Precision (%)	Recall (%)	F1 score (%)	mAP@0.5 (%)	mAP@0.5:0.95 (%)	Model size (MB)	Parameters (M)	GFLOPs
✓					72.66	75.72	74.16	76.17	66.11	5.58	2.45	8.5
✓	✓				72.43	76.56	74.44	77.25	67.74	5.76	2.56	9.81
✓	✓	✓			74.64	74.49	74.56	78.39	67.19	5.81	2.57	10.56
✓	✓	✓	✓		72.37	76.15	74.21	77.68	66.79	5.64	2.48	10.38
✓	✓	✓	✓	✓	72.65	76.08	74.33	77.45	66.96	5.69	2.50	10.72

**Table 5:** Class-wise detection performance of YOLOv26n-OBB with proposed modules.

Model					Ripe fruit		Half-ripe fruit		Unripe fruit		Stem	
YOLOv26n-OBB	FFLM	SDEM	GSEConv	EUCB	mAP@0.5 (%)	mAP@0.5:0.95 (%)	mAP@0.5 (%)	mAP@0.5:0.95 (%)	mAP@0.5 (%)	mAP@0.5:0.95 (%)	mAP@0.5 (%)	mAP@0.5:0.95 (%)
✓					86.54	81.59	77.12	75.35	79.87	67.12	61.15	40.38
✓	✓				85.36	82.14	79.46	75.49	81.04	72.32	63.14	41.01
✓	✓	✓			86.44	82.11	79.12	74.65	84.89	69.62	63.11	42.38
✓	✓	✓	✓		85.15	81.56	78.41	73.45	83.89	71.09	63.27	41.06
✓	✓	✓	✓	✓	85.26	83.12	78.11	73.42	83.41	69.81	63.02	41.49

The baseline YOLOv26n-OBB achieves 76.17% mAP@0.5 and 66.11% mAP@0.5:0.95. After introducing the Feature Fusion Lite Module (FFLM), mAP@0.5 and mAP@0.5:0.95 increase to 77.25% and 67.74%, respectively, indicating that cross-layer feature fusion can improve feature representation in the YOLOv26n-OBB framework. With the further incorporation of SDEM, mAP@0.5 increases to 78.39%, although mAP@0.5:0.95 slightly decreases to 67.19%. This suggests that SDEM improves detection performance, while its effect on stricter localization metrics is relatively limited.

The class-wise results in **Table 5** further show that the proposed modules have different effects on different categories. For the stem category, mAP@0.5 increases from 61.15% to 63.14% after introducing FFLM, reflecting improved sensitivity to slender structural targets. After SDEM is further added, the stem mAP@0.5 remains almost unchanged, while mAP@0.5:0.95 increases from 41.01% to 42.38%, indicating improved localization quality for stem detection. For fruit-related categories, the improvements are not uniform across all metrics, suggesting that different modules contribute differently to feature representation under occlusion and scale variation.

When GSEConv and EUCB are sequentially integrated, the model maintains competitive accuracy with a moderate increase in computational cost. The final configuration achieves 77.45% mAP@0.5 and 66.96% mAP@0.5:0.95. Although this configuration does not yield the highest accuracy among all variants, it provides a more balanced trade-off between accuracy, model size, parameter count, and computational cost, which is desirable for deployment in resource-constrained robotic systems.

Overall, the experimental results demonstrate that the proposed modules can be transferred to a more recent lightweight OBB detector such as YOLOv26n-OBB. In addition, different module combinations exhibit different accuracy-efficiency trade-offs, indicating that the optimal configuration should be selected according to specific application requirements.

### 3.5 Evaluation of fruit-stem association and picking point localization

#### 3.5.1 Fruit-stem association results

**Figure 11:**
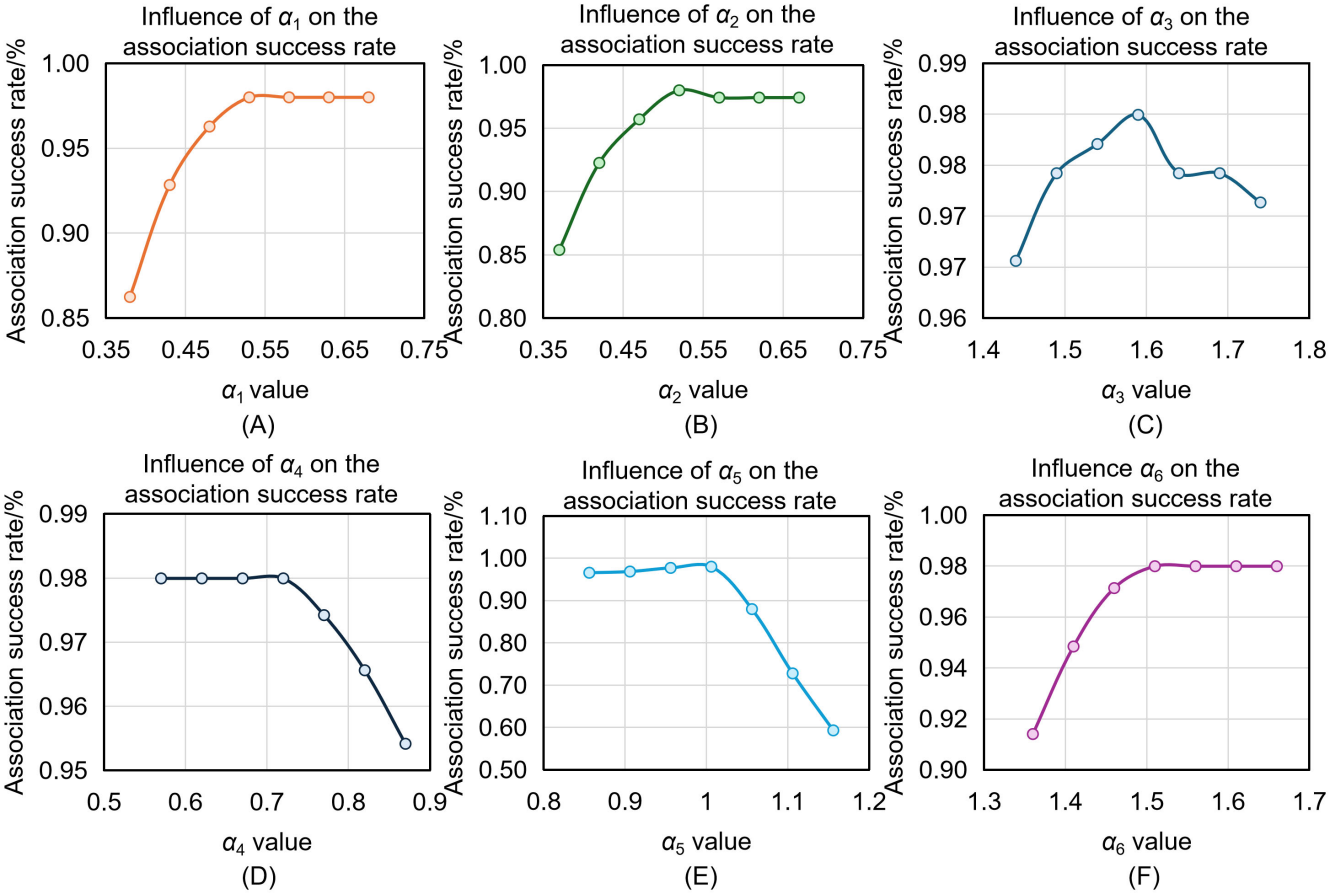
Single-factor sensitivity analysis of the six association parameters on the fruit-stem association success rate. **(A)**. Influence of *α*_1_ on association success rate; **(B)**. Influence of *α*_2_ on association success rate; **(C)**. Influence of *α*_3_ on association success rate; **(D)**. Influence of *α*_4_ on association success rate; **(E)**. Influence of *α*_5_ on association success rate; **(F)**. Influence of *α*_6_ on association success rate.

To further evaluate the robustness of the selected parameters, a single-factor sensitivity analysis was conducted using 165 strawberry images from the test set. During the sensitivity analysis, only one parameter was changed at a time with a step size of 0.05, while the remaining parameters were kept fixed at their calibrated values. The fruit-stem association success rate was used as the evaluation metric. The experimental results are shown in **Figure 11**. The results indicate that the association success rate is sensitive to several parameters, especially the lower bound of the projection-ratio constraint.

For *α*_1_, the association success rate increased from 86.25% to 97.99% as the value increased from 0.38 to 0.53, and then remained stable when *α*_1_ was further increased. This indicates that *α*_1_ = 0.53 is sufficient to include most correct fruit-stem pairs while avoiding an overly loose endpoint-distance constraint. A similar trend was observed for *α*_2_. When *α*_2_ increased from 0.37 to 0.52, the association success rate increased from 85.39% to 97.99%. Further increasing *α*_2_ did not improve the association performance and slightly reduced the success rate to 97.42%, indicating that *α*_2_ = 0.52 provides a suitable balance between tolerance to lateral deviation and suppression of incorrect matches. For *α*_3_, the association success rate reached the highest value of 97.99% at *α*_3_ = 1.59. Smaller values tended to reject some true fruit-stem pairs because of viewpoint-induced spatial variation, whereas larger values slightly reduced the discriminative ability of the center-distance constraint. For *α*_4_, the association success rate remained 97.99% when *α*_4_ ranged from 0.57 to 0.72, but gradually decreased when *α*_4_ was further increased. This result suggests that an excessively strict direction constraint may reject valid pairs with curved, tilted, or partially occluded stems. Therefore, *α*_4_, obtained from the percentile-based calibration, was selected because it maintains the highest association success rate while preserving sufficient orientation discrimination. The lower-bound projection coefficient *α*_5_ showed the highest sensitivity. The association success rate reached 97.99% at *α*_5_ = 1.006, but decreased sharply to 87.97%, 72.78%, and 59.31% when *α*_5_ was increased to 1.056, 1.106, and 1.156, respectively. This indicates that many correct stem lower endpoints are located very close to the fruit upper endpoint in the projected image. Therefore, setting *α*_5_ too high would incorrectly exclude a large number of true fruit-stem associations. For the upper-bound projection coefficient *α*_6_, the association success rate increased from 91.40% to 97.99% as *α*_6_ increased from 1.36 to 1.51, and then remained stable. Thus, *α*_6_ = 1.51 was selected as the smallest value that achieved the highest association success rate, preventing the projection constraint from becoming unnecessarily loose.

Overall, the sensitivity analysis demonstrates that the selected parameter combination (*α*_1_, *α*_2_, *α*_3_, *α*_4_, *α*_5_, *α*_6_) = (0.53, 0.52, 1.59, 0.72, 1.006, 1.51) achieves the highest fruit-stem association success rate of 97.99%. The results also show that the selected parameters are located within stable or optimal intervals, confirming the rationality of the percentile-based parameter calibration strategy. The failed association cases were mainly related to severe stem occlusion, incomplete stem detection, or inaccurate estimation of the stem lower endpoint. In these cases, the geometric relationship between the fruit and stem could not be fully captured by the OBBs, which affected the subsequent association judgment.

**Figure 12:**
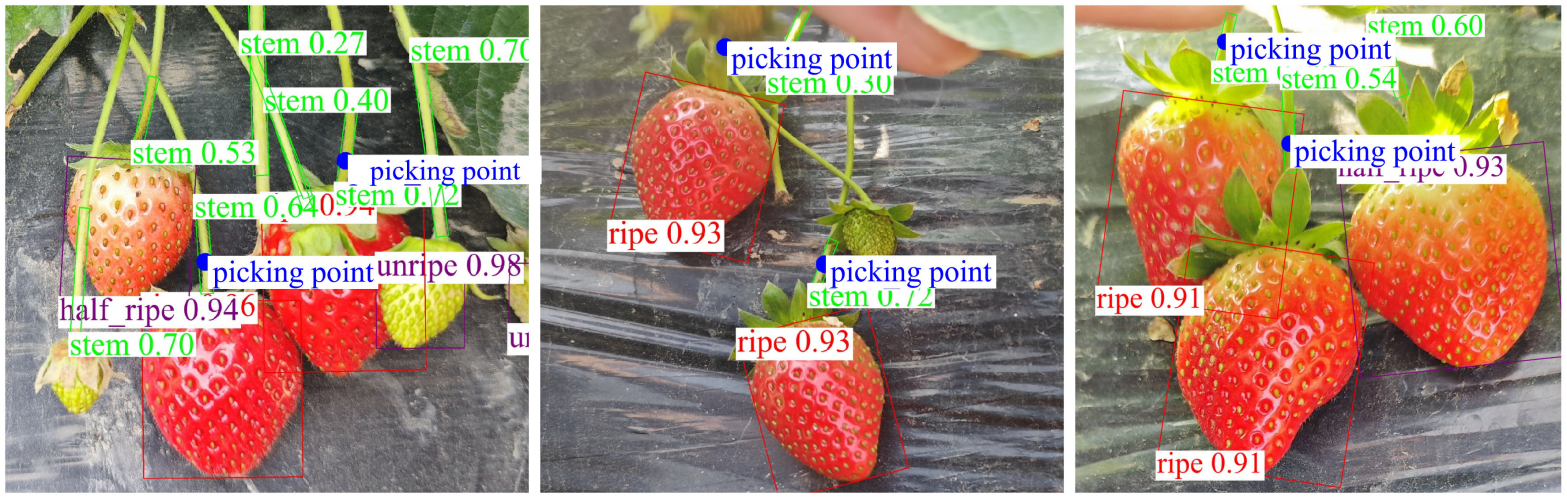
Visualization of fruit-stem association and picking point localization.

In this section, the picking points shown in **Figure 12** were determined according to the geometric size of strawberries in the image. An approximate pixel-to-physical scale was estimated, and the corresponding pixel coordinates of the picking points were calculated accordingly. These coordinates were used only for two-dimensional visualization of the fruit-stem association and picking point localization results. In the subsequent 3D localization experiment, a depth camera was used to acquire the predicted 3D coordinates of the picking points in the depth camera coordinate system through depth back-projection based on aligned RGB-D data and camera intrinsic parameters.

#### 3.5.2 Accuracy evaluation of picking point localization

In the picking point localization experiment, the predicted picking point coordinates were obtained in the depth camera coordinate system, while the ground-truth coordinates were determined through manual calibration. The localization accuracy was evaluated by calculating the absolute errors between the predicted and ground-truth coordinates.

The localization results are presented in **Table 6**. In the *x-*, *y-*, and *z-*directions, the maximum absolute localization errors were 2.6 mm, 3.3 mm, and 3.1 mm, respectively, while the corresponding average absolute localization errors were 2.2 mm, 2.6 mm, and 2.3 mm. These preliminary results indicate that the proposed localization method has the potential to support stem-cutting point estimation for strawberry harvesting robots.

**Table 6:** Predicted coordinates, ground-truth coordinates, and absolute localization errors of picking points.

Experiment no.	Predicted picking point coordinates (mm)			Ground-truth coordinates(mm)			Absolute localization errors (mm)		
	*X_pk_*	*Y_pk_*	*Z_pk_*	*X_gk_*	*Y_gk_*	*Z_gk_*	*x*	*y*	*z*
1	-31.1	34.1	307.4	-33.5	31.4	304.7	2.4	2.7	2.7
2	-37.5	39.4	287.1	-34.9	36.1	289.3	2.6	3.3	2.2
3	-74.2	45.4	323.5	-71.7	47.9	324.1	2.5	2.5	0.6
4	-45.1	17.7	284.5	-43.4	15.2	281.7	1.7	2.5	2.8
5	-27	34.4	285.4	-25.4	31.4	287.8	1.6	3.0	2.4
6	-20.8	-14.1	272.8	-23.1	-16.2	275.1	2.3	2.1	2.3
7	-24.4	53.9	272	-26.8	51.4	274.5	2.4	2.5	2.5
8	-31	-23.7	297.1	-33.3	-21.1	294.5	2.3	2.6	2.6
9	-62.3	34.6	295.7	-60.1	32.5	293.8	2.2	2.1	1.9
10	-35.9	-98.6	293.4	-38.1	-95.7	296.1	2.2	2.9	2.7
11	-68.2	47.5	299.5	-65.8	44.6	302.6	2.4	2.9	3.1
12	-41.7	35.4	311.7	-39.4	37.6	309.4	2.3	2.2	2.3
Average							2.2	2.6	2.3

**Figure 13:**
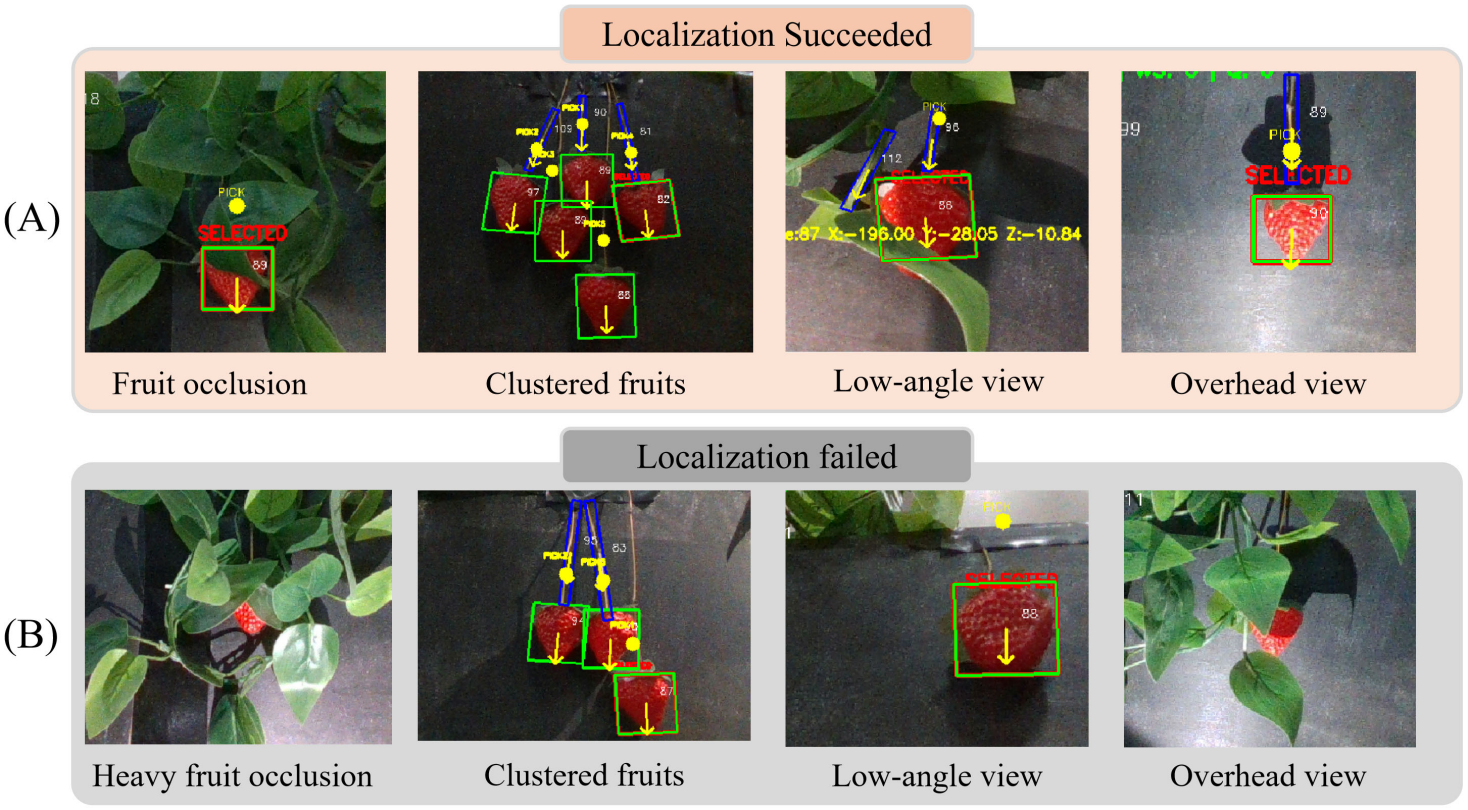
Qualitative picking point localization results under non-ideal harvesting conditions. **(A)** Successful localization cases. **(B)** Representative failure localization cases.

To further evaluate the robustness of the proposed method in realistic harvesting scenarios, additional qualitative tests were conducted under several non-ideal conditions, including fruit occlusion, heavy fruit occlusion, clustered fruits, low-angle views, and overhead views. The corresponding results are shown in **Figure 13**.

As shown in **Figure 13A**, the proposed method can still achieve reasonable localization results under moderately challenging conditions. In the fruit occlusion case, although part of the strawberry is covered by leaves, the target fruit can still be detected and a valid picking point can be generated. In the clustered-fruit case, multiple strawberries appear close to each other, resulting in adjacent or overlapping fruit regions. The proposed fruit-stem geometric association strategy helps determine the target fruit and its corresponding stem, thereby reducing the risk of picking point estimation based only on fruit morphology. For the low-angle and overhead views, the method can still generate valid localization results when the fruit and stem remain sufficiently visible.

However, **Figure 13B** shows that the method may fail under more severe conditions. In the heavy fruit occlusion case, the strawberry or its peduncle is largely covered by leaves, which makes it difficult to obtain reliable fruit-stem detection results. In dense clustered scenes, several fruits and stems may overlap in a small image region, leading to ambiguous fruit-stem correspondence. In the low-angle view, the projected geometric relationship between the fruit and stem may be distorted, which can cause the estimated picking point to deviate from the true fruit-stem junction. In the overhead view, leaves and the fruit body may obscure the peduncle, resulting in missed stem detection or failure to generate a valid picking point.

Overall, these additional results demonstrate that the proposed method has a certain degree of robustness under moderate occlusion, clustered fruit distribution, and non-frontal viewpoints. Nevertheless, its performance still decreases when the peduncle is severely occluded, invisible, or inaccurately detected. These failure cases indicate that reliable stem perception remains a key challenge for robotic strawberry harvesting in complex field environments.

#### 3.5.3 Simulated harvesting results

The experimental results, including picking point localization time, total harvesting time, and simulated harvesting success rate for each trial, were further averaged (**Table 7**). The average picking point localization time for a single strawberry was 1.2 s, while the average total harvesting time per fruit was 9.2 s. In addition, 56 out of 60 simulated harvesting attempts were successful, resulting in a simulated harvesting success rate of 93.3%.

**Table 7:** Results of the simulated harvesting experiment.

Trial	Average picking point localization time per fruit (s)	Average harvesting time per fruit (s)	Simulated harvesting success rate (%)
1	1.2	9.2	90.0
2	1.1	9.1	95.0
3	1.3	9.3	95.0
Average	1.2	9.2	93.3

These results indicate that the proposed FSGE-OBB-based perception framework can provide stable and timely picking point localization, while the geometric fruit-stem association strategy ensures reliable correspondence under typical harvesting conditions. Combined with the robotic execution system, the overall pipeline achieves efficient closed-loop harvesting performance, demonstrating the feasibility of the proposed method for automated strawberry harvesting tasks in simulated environments.

Failure cases were primarily attributed to two factors. First, the use of artificially substituted stem materials, which were thinner and less structurally consistent than real strawberry stems, degraded stem detection reliability and occasionally led to missed or inaccurate stem localization. Second, severe occlusion of fruits or stems in dense scenes resulted in incomplete detection outputs, which further propagated errors into the fruit-stem association stage and subsequently caused harvesting failures. These limitations collectively account for the observed reduction in harvesting success rate.

### 3.6 Discussion

Although the proposed method demonstrated promising performance in the simulated robotic harvesting experiment, its methodological contribution should be further clarified by comparison with existing strawberry picking point localization approaches. As summarized in **Table 8**, existing methods can be broadly categorized into keypoint detection-based methods, HBB-based methods, segmentation-based methods, and fruit OBB detection-based methods. The proposed fruit-stem OBB association-based method is also included in the table for comparative analysis.

**Table 8:** Comparison between the proposed method and existing strawberry picking point localization methods.

Method type	Main idea	Typical technique	Strengths	Limitations
Keypoint detection-based methods	Directly predict picking points	YOLO-Pose, keypoint networks	High localization accuracy when fruit-stem structures are visible	Sensitive to occlusion and incomplete stem visibility
HBB-based methods	Infer picking points from fruit or fruit-stem horizontal boxes	YOLO, Faster R-CNN	Simple task design and low model complexity	Object orientation is not explicitly represented; HBBs cannot accurately describe inclined fruits or slender stems
Segmentation-based methods	Use fruit masks, contours, or peduncle-related regions for localization	Mask R-CNN, YOLO-seg	Fruit shape, contour, or peduncle-related information can be obtained	Reliable stem or peduncle segmentation remains difficult under occlusion and dense canopy conditions
Fruit OBB detection-based methods	Estimate picking points from fruit oriented boxes	YOLO-OBB-based detectors	Fruit orientation can be represented	Stem orientation and fruit-stem correspondence are ignored
Proposed method	Detect fruit and stem OBBs and localize picking points based on their spatial relationship	FSGE-OBB + geometric association	Explicitly models fruit-stem structure and supports 3D picking point localization	Depends on stem detection and calibrated geometric parameters

Keypoint detection-based methods can directly predict picking points and usually achieve high localization accuracy when the fruit-stem region is clearly visible. However, their performance may degrade under severe occlusion or incomplete stem visibility. HBB-based methods are simple and computationally efficient, but horizontal boxes cannot explicitly represent object orientation; therefore, they are limited in describing inclined fruits and slender stems in complex harvesting scenes. Segmentation-based methods can provide fruit masks, contours, or peduncle-related shape information for localization, but reliable segmentation of stems or peduncles remains challenging under occlusion and dense canopy conditions. Fruit OBB detection-based methods can describe fruit orientation more effectively than HBB-based methods, but they still rely mainly on fruit geometry and do not explicitly establish the correspondence between fruits and stems.

Compared with these methods, the proposed method jointly detects ripe fruits and stems using oriented bounding boxes and further establishes their geometric association for picking point localization. Therefore, the picking point is determined based on the spatial relationship between the target fruit and its corresponding stem, rather than only relying on fruit position, fruit morphology, segmentation masks, or predefined keypoints. This design is more suitable for stem-cutting strawberry harvesting tasks, where the picking point is closely related to the fruit-stem junction.

Nevertheless, several limitations remain. First, the proposed method still depends on reliable stem detection and calibrated geometric parameters. Its performance may decrease when strawberries are heavily occluded, peduncles are invisible or only partially visible, multiple fruits are densely clustered, or images are captured from less favorable viewpoints. In these cases, severe occlusion or incomplete stem detection may lead to unreliable stem endpoint estimation, incorrect fruit-stem association, and inaccurate picking point localization. Second, dense fruit clusters may introduce ambiguity in fruit-stem correspondence, especially when multiple fruits and stems overlap or appear close to each other. Although the proposed geometric constraints can reduce unreasonable matches, incorrect associations may still occur in highly clustered scenes. Third, low-angle or overhead views may distort the projected fruit-stem geometry and weaken the reliability of the association constraints, resulting in picking point deviation or localization failure.

An additional practical challenge is the domain shift between the smartphone-acquired RGB dataset used for model training and the D435i-acquired RGB-D data used in the robotic localization experiment. Differences in field of view, lens distortion, exposure, color response, and image noise may influence detection performance when the model is transferred to the D435i-based harvesting platform. In this study, an independent D435i-acquired detection test set was not constructed. Therefore, the D435i-based experiments should be regarded as preliminary validation of the picking point localization and simulated harvesting pipeline. Accordingly, the reported detection metrics should be interpreted as performance on the smartphone-acquired RGB test set rather than direct detection performance on D435i-acquired images.

For practical robotic harvesting deployment, real environments may involve more complex illumination, plant motion, depth noise, dense canopy structures, and severe occlusion. These factors may further affect the stability of detection, fruit-stem association, and 3D localization. Future work will focus on collecting larger-scale RGB-D datasets in real ridge-cultivated strawberry harvesting environments, improving stem perception under severe occlusion, incorporating multi-view or temporal information, and validating the complete robotic harvesting system under real greenhouse or field conditions.

## 4 Conclusions

To address the challenges of large-scale differences between strawberry fruits and stems, severe stem occlusion, missed detections of small targets, and picking point localization in ridge-cultivated environments, this study presents a fruit-stem oriented detection and picking point localization method based on an improved YOLOv11n-OBB model. The main conclusions are summarized as follows:

1. An oriented bounding box dataset for fruit–stem detection was constructed under ridge-cultivated strawberry field conditions. The dataset includes 1,652 valid images and four categories—ripe fruit, half-ripe fruit, unripe fruit, and stem, covering diverse lighting conditions, occlusion levels, and maturity stages, thereby providing reliable data support for fruit-stem oriented detection in complex field environments.
2. An improved oriented object detection model, termed FSGE-OBB, was developed based on YOLOv11n-OBB. By enhancing cross-scale feature interaction, directional representation of slender stems, and high-resolution detail recovery through lightweight module design, the proposed model achieves mAP@0.5 and mAP@0.5:0.95 values of 78.85% and 68.27%, respectively, representing improvements of 2.59 and 1.90 percentage points over the baseline. The Recall reaches 77.02%, indicating improved detection performance for small and slender targets in complex field conditions.
3. A geometry-constrained fruit-stem association method was established based on the spatial relationship between fruit and stem OBBs. The proposed method achieves a success rate of 97.99% on the test set, demonstrating that geometric constraints can effectively determine fruit–stem correspondence. However, severe occlusion and incomplete stem detection may still affect association accuracy.
4. A picking point localization strategy was developed for different detection scenarios. When both fruit and stem are detected, the picking point is localized along the stem center-line segment direction; when only the fruit is detected, the picking point is estimated based on the fruit center-line segment. In a preliminary 3D localization experiment involving 12 ripe fruits, the mean absolute errors in the *x-*, *y-*, and *z-*directions were 2.2 mm, 2.6 mm, and 2.3 mm, respectively, indicating the potential effectiveness of the proposed method for stem-cutting point estimation. In addition, simulated harvesting experiments on a self-developed three-axis strawberry harvesting robot achieved a harvesting success rate of 93.3%, indicating that the proposed method can satisfy the basic operational requirements of robotic strawberry harvesting.

Overall, the proposed method integrates oriented object detection, fruit-stem geometric association, and depth-based 3D localization into a unified framework, providing a feasible visual perception solution for strawberry harvesting robots in ridge-cultivated environments. Future work will focus on collecting larger-scale RGB-D datasets, improving robustness under severe occlusion, optimizing embedded deployment, and validating the proposed method in real robotic harvesting scenarios.

## Data Availability

The original contributions presented in the study are included in the article/supplementary material. Further inquiries can be directed to the corresponding authors.
